# Taxonomy and species-groups of the subgenus *Crematogaster* ( *Orthocrema*) in the Malagasy region (Hymenoptera, Formicidae)

**DOI:** 10.3897/zookeys.199.2631

**Published:** 2012-06-04

**Authors:** Bonnie B. Blaimer

**Affiliations:** 1 Department of Entomology, University of California-Davis, One Shields Ave, Davis, CA 95616, USA

**Keywords:** *Crematogaster*, *Orthocrema*, Madagascar, Indian Ocean islands, taxonomy, intermediate workers

## Abstract

The species-level taxonomy of the subgenus *Crematogaster* (*Orthocrema*) in the Malagasy region is evaluated with both morphological data from worker and queen ants, and genetic data from three nuclear markers (long wavelength rhodopsin, arginine kinase and carbamoylphosphate synthase). These two types of data support the existence of six *Orthocrema* species: *Crematogaster madecassa* Emery, *Crematogaster rasoherinae* Forel, *Crematogaster telolafy* **sp. n.**, *Crematogaster razana* **sp. n.**, *Crematogaster volamena* **sp. n.** and *Crematogaster mpanjono* **sp. n.**. Two new synonyms of *Crematogaster rasoherinae* Forel are recognized, *Crematogaster rasoherinae brunneola* Emery, **syn.** **n.** and *Crematogaster voeltzkowi* Forel, **syn. n.**, as these were not supported as distinct taxa by the data. A neotype is designated for *Crematogaster rasoherinae*; lectotypes are designated for *Crematogaster madecassa*, *Crematogaster rasoherinae brunneola* and *Crematogaster voeltzkowi*. Species descriptions, images, distribution maps and identification keys based on worker and queen ants are given for all six species. A diagnosis of the subgenus *Orthocrema* in the Malagasy region is presented for both workers and queens. Within the Malagasy *Orthocrema*, three distinct phylogenetic lineages are suggested by molecular and morphological data. Newly defined monophyletic species-groups are thus the *Crematogaster madecassa*-group (*Crematogaster madecassa*, *Crematogaster telolafy* and *Crematogaster razana*) and the *Crematogaster volamena*-group (*Crematogaster volamena* and *Crematogaster mpanjono*); *Crematogaster rasoherinae* represents an isolated lineage in the Malagasy region and its closest relatives remain unclear. Other interesting biological findings are the presence of an intermediate caste between workers and queens in *Crematogaster rasoherinae* and *Crematogaster madecassa*, and unusually large workers in *Crematogaster volamena* resembling a major caste.

## Introduction

The large and species-rich ant genus *Crematogaster* Lund (> 450 described species; Bolton 2011) has a global distribution throughout most forest and savannah habitats in warm-temperate to subtropical and tropical climates. *Crematogaster* species diversity is highest in the tropics and subtropics however, where these ants often form a dominant part of the local ant community. The majority of *Crematogaster* species nest arboreally, for example in twigs or under bark, but ground nesting seems to be equally common in temperate and cooler climates and also more prevalent in some species-groups in the tropics. The species-level taxonomy of *Crematogaster* ants is notoriously difficult and encumbered by synonyms and ambiguous subspecies names (Longino 2003; Ward 2010). Blaimer (2010) provided a comprehensive review on the natural history and taxonomic state of *Crematogaster*.

In the Malagasy region, here defined as Madagascar and the surrounding Indian Ocean islands, the taxonomy of *Crematogaster* is currently being revised in a series of publications, subdivided into the several distinct species-groups present in the region (see Blaimer 2010, 2012). The total species diversity of *Crematogaster* in the Malagasy region is estimated to be approximately 33 species (Blaimer, unpublished data). This estimate represents a mixture of previously described species, species new to science, and reductions due to synonymy. Most of these species occur only in Madagascar, but five species also are found on the Comoros Islands, Mayotte and the Seychelles. Recent intensive inventories of arthropods and especially the ant fauna in the Malagasy region (see e.g. Fisher and Penny 2008) have immensely increased the extent of available specimens for revisionary work, generating much more complete distribution records for already described species and discovering numerous undescribed new species.

The present study is part of this larger revisionary work and treats all species associated with the subgenus *Orthocrema* Santschi (1918) in the Malagasy region. Recent molecular work (Blaimer, in prep.) has found Malagasy species placed in the subgenus *Mesocrema* Santschi (1928) to be closely related to the former, and these are therefore included with *Orthocrema* in the present revision. This altered classification follows anticipated changes in the subgeneric classification of *Crematogaster* in the near future, based upon a molecular phylogenetic framework (Blaimer in prep.).

Up to now, one species has been described from the Malagasy region for *Orthocrema*, *Crematogaster madecassa* Emery, whereas three species and subspecies have been described for *Mesocrema*: *Crematogaster rasoherinae* Forel, *Crematogaster rasoherinae brunneola* Emery and *Crematogaster voeltzkowi* Forel. The latter has been recorded exclusively from the Comoros Islands, whereas the other species were first described from Madagascar. My observations suggest that the Malagasy *Orthocrema* present an exception to the predominantly arboreal life habit of *Crematogaster* ants in this region. Most species in this group appear to be generalists, as they have been collected nesting both on the ground in rotten logs or branches, or arboreally in dead twigs or bark and canopy moss mats. A very interesting aspect of the biology of some of the Malagasy species in this group is the presence of intermediate workers in the colony. These possess morphological features that are intermediate between workers and queens, but their function and behavior in the colony remains unclear. Intermediates have also been reported in the North American *Crematogaster* (*Orthocrema*) species *Crematogaster smithi*,where they were denoted as ‘large workers’ (Heinze et al. 1999). In the case of the latter, it was shown that these had the ability to lay unfertilized trophic eggs, but were not capable of sexual reproduction (Heinze et al. 1999, 2000). The presence of this separate caste may be a more widespread phenomenon in *Orthocrema* species.

In the following, I focus on a reevaluation of the presently described Malagasy *Orthocrema* species with both morphological and molecular methods, and further describe new species that are supported by these two types of data. A second aim of this study is the delimitation of two morphologically and genetically distinct species-groups within Malagasy *Orthocrema*.

## Materials

### Morphological study

All morphological observations were made with a Leica MZ12.5 stereomicroscope. Standard measurements (in mm) were taken at 50× with a Wild M5A stereomicroscope and a dual-axis Nikon micrometer wired to a digital readout. Measurements are given to the second decimal place, and indices are presented as decimal fractions (also to the second decimal). Ranges are always expressed as minimum – maximum values. Measured specimens were chosen to represent the entire distribution range of a given species. The abbreviations used for measurements and indices below follow Blaimer (2010) and Longino (2003); for illustrations of these see Blaimer (2010).

### Measurements and indices

**HW** Maximum head width including eyes, in full face view.

**HL** Head length; perpendicular distance from line tangent to rearmost points of vertex margin to line tangent to anterior most projections of clypeus, in full face view.

**EL** Eye length; measured along the maximum diameter.

**SL** Scape length; length of scape shaft from apex to basal flange, not including basal condyle and neck. If scape is strongly arched, this measurement is taken as the chord length from the basal flange to the apex.

**PTL** Petiole length; measured in lateral profile as the distance from dorsoposterior margin of segment to anterior inflection point where petiole curves up to condyle.

**PTH** Petiole height; measured in lateral profile as vertical distance from ventral margin to highest point of dorsoposterior margin.

**PTW** Petiole width; maximum width of petiole in dorsal view.

**PPL** Postpetiole length; measured in dorsal view at an angle that maximizes length.

**PPW** Postpetiole width; measured in same view as and perpendicular to postpetiole length.

**WL** Weber’s length; measured in lateral profile of mesosoma, distance from approximate inflection point, where downward sloping pronotum curves into anteriorly projecting neck, to ventroposterior propodeal lobes.

**SPL** Propodeal spine length; measured from tip of propodeal spine to closest point on outer rim of propodeal spiracle, maximizing spine length in lateral view.

**LHT** Length of metatibia, excluding the proximomedial condyle.

**CI** Cephalic index: HW/HL.

**OI** Ocular index: EL/HL.

**SI** Scape index: SL/HW.

**PTHI** Petiole height index: PTH/PTL.

**PTWI** Petiole width index: PTW/PTL.

**PPI** Postpetiole width index: PPW/PPL.

**SPI** Propodeal spine index: SPL/WL.

**LBI** Leg-body index: WL/LHT.

### Queen-specific measurements:

**MSNW** Mesonotal width; maximum width of mesonotum, measured in dorsal view.

**MSNL** Mesonotal length; maximum length of mesonotum, measured in dorsal view.

**MSNI** Mesonotal index: MSNW/MSNL.

Color images were created with a JVC KY-F75U digital camera, a Leica MZ16A stereomicroscope and ZERENE STACKER (v1.02) software. The scanning electron microscope images were taken at the California Academy of Sciences using a Zeiss/LEO 1450VP SEM. All ant images presented here are also publicly available on AntWeb (www.antweb.org). Line drawings were produced by tracing color images in Adobe Illustrator CS5.Species distributions were plotted with ARCMAP (v9.3) within the software ARCGIS, based on coordinates (latitude and longitude) as given on the specimen labels of all material (see also supplementary table 1 for a species list with GPS coordinates). For material lacking this information, i.e. syntype specimens, the following sources were used to georeference collection sites: the GEOnet Names Server (National Geospatial-Intelligence Agency 2010) and the Gazetteer to Malagasy Botanical Collecting Localities (Schatz and Lescot 2003). Classification of major geographic regions in Madagascar throughout species descriptions follows Gautier and Goodman (2003). Common abbreviations within locality data are: P.N. = Parc National, R.S. = Réserve Spéciale, F = Forêt, P.C. = Parc Naturel Communautaire, R.N.I. = Réserve Naturelle Intégrale.

**Table 1.** **Specimen data and GenBank accessions.** Information on vouchers, GenBank accession numbers and locality data on all specimens included in the molecular analyses.

**Taxon**	**Voucher**	**GenBank accession**			**Collection locality**	**LatDD, LongDD**
***Crematogaster***		**LW Rh**	**ArgK**	**CAD**		
*madecassa*_amdi	CASENT0068164	JQ326949	JQ326913	JQ326932	Madagascar: Toamasina: Res. Ambodiriana, 4.8 km 306°Manompana, 125m	-16.672, 49.701
*madecassa*_mjy	CASENT0525407	JQ326950	JQ326914	JQ326933	Madagascar: Antsiranana: P.N. Marojejy, Manantenina River, 27.6 km 35° NE Andapa, 775m	-14.435, 49.760
*mpanjono*_man	CASENT0193212	JQ326943	JQ326909	JQ326937	Madagascar: Antsiranana: R.S. Manongarivo, 10.8 km 229° SW Antanambao, 400m	-13.962, 48.433
*mpanjono*_nb	CASENT0056947	JQ326947	JQ326910	JQ326929	Madagascar: Antsiranana: Nosy-Be: Antsirambazaha, Hell-Ville, 143m	-13.413, 48.311
*razana*_kal	CASENT0193589	JQ326952	JQ326915	JQ326938	Madagascar: Toliara: RS Kalambatritra, 1365m	-23.419, 46.458
*razana*_tsi	CASENT0193591	JQ326954	JQ326916	JQ326939	Madagascar: Toliara: P.N. Andohahela, F d’Ambohibory, 1.7 km 61° ENE Tsimelahy,300m	-24.930, 46.646
*telolafy*_isa	CASENT0492527	JQ326951	JQ326917	JQ326935	Madagascar: Fianarantsoa: Parc National d’Isalo, 29.2 km 351° N Ranohira, 500m	-22.313, 45.292
*volamena*_aza	CASENT0193590	JQ326945	JQ326911	JQ326930	Madagascar: Antsiranana: 6.9 km NE Ambanizana, Ambohitsitondroina, 825m	-15.567, 50.000
*volamena*_vaky	CASENT0162194	JQ326946	JQ326912	JQ326931	Madagascar: Toamasina: RS Ambatovaky, Sandrangato river, 400m	-16.817, 49.293
*rasoherinae*_maha	CASENT0070841	JQ326941	JQ326922	JQ326941	Madagascar: Fianarantsoa: R.F. Agnalazaha, Mahabo, 42.9 km 215° Farafangana, 20m	-23.194, 47.723
*rasoherinae*_ahe	CASENT0193412	JN129958	JN129923	JN129882	Madagascar: Toliara: P.N. Andohahela/parcel 3; near Forest station; 3.9km Ranopiso, 170m	-25.018, 46.652
*rasoherinae*_koe	CASENT0487673	JQ326942	JQ326921	JQ326942	Madagascar: Antsiranana: Forêt d’ Andavakoera, 21.4km 75° ENE Ambilobe, 425m	-13.118, 49.230
*rasoherinae*_com	CASENT0147455	JQ326953	JQ326920	JQ326925	Comoros: Anjouan: Hajoho, 10m	-12.122, 44.488
*sordidula*	CASENT0193797	JQ326944	JQ326919	JQ326944	Croatia: N Dalmatia: Pakoštane, 40m	43.917, 15.500
*longipilosa*	CASENT0193780	JQ326948	JQ326918	JQ326934	Malaysia: Selangor: Ulu Gombak, 330m	3.300, 101.783
*cf_dolens*	CASENT0193756	JQ326956	JQ326923	JQ326940	Kenya: Western Prov.: Arabuko Sokoke Forest, 10m	-3.325, 39.948
*arcuata*	CASENT0193084	JQ326955	JQ326924	JQ326936	Venezuela: Aragua: Estacion Rancho-Grande, PN Henri Pittier, 1100m	10.582, -68.474

The International Commission on Zoological Nomenclature (1999) requires lectotypes designated after 1999 to “contain an express statement of deliberate designation” (amended Article 74.7.3). I use the statement ‘lectotype by present designation’ to fulfill this requirement. Lectotypes have been designated where a name lacks a holotype or lectotype and unambiguous syntypes have been identified. The purpose is to provide stability of nomenclature, and designation is done in a revisionary context in agreement with the amended Recommendation 74G of Article 74.7.3. Neotype designations have further been made for names with no extant name-bearing types that are in need of a name-bearing type “to objectively define the nominal taxon” (Article 75.1, ICZN, 1999), and are in agreement with the qualifying conditions stated in Article 75.3 (ICZN, 1999).

### Specimens were examined and/or deposited in the following collections:

**CASC** California Academy of Sciences, San Francisco, CA, USA

**BBBC** B.B. Blaimer Collection, University of California at Davis, CA, USA

**MCZC** Museum of Comparative Zoology, Harvard, USA

**MHNG** Muséum d’Histoire Naturelle, Genève, Switzerland

**MSNG** Museo Civico di Storia Naturale, Genova, Italy

**NHMB** Naturhistorisches Museum, Basel, Switzerland

**PSWC** P.S. Ward Collection, University of California at Davis, CA, USA

**SAMC** South African Museum, Cape Town, South Africa

**ZMBH** Museum für Naturkunde der Humboldt Universität, Berlin, Germany

### Molecular data collection and phylogenetic analyses

After sorting all available specimens to morphospecies, one to four individual worker ants for each of six putative Malagasy *Crematogaster* (*Orthocrema*) species were selected for genetic analysis. Four non-Malagasy *Orthocrema* species were chosen as outgroups, given their approximate relationships to the Malagasy taxa as known from a previous, larger phylogenetic analysis (Blaimer, in prep.). Two of these (*Crematogaster sordidula* Nylander and *Crematogaster longipilosa* Forel) represent distant relatives to all Malagasy *Orthocrema*, whereas the remaining two taxa (*Crematogaster arcuata* Forel and *Crematogaster cf. dolens* Forel) are closer relatives to the Malagasy taxa. For the distribution of the sampled taxa refer to Table 1.

**Figure 1.**
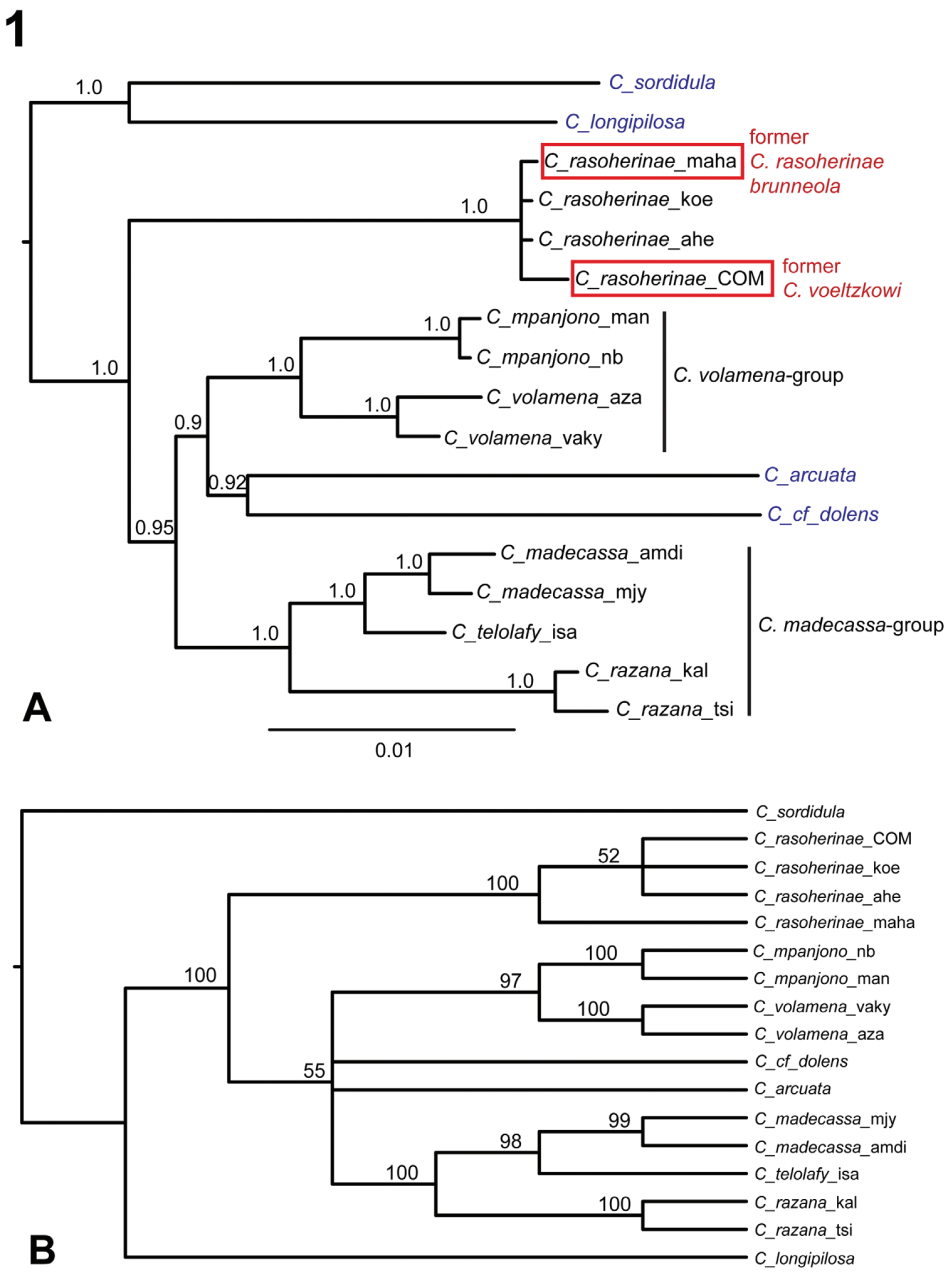
Species phylogeny of Malagasy *Crematogaster* (*Orthocrema*). **A** Results of Bayesian inference summarized as consensus tree in MrBayes. Support values on branches represent posterior probabilities; scalebar shows nucleotide changes per base pair. Newly defined species-groups, and the specimen representing former *Crematogaster voeltzkowi* are indicated. Outgroup species are marked by blue font **B** ML-consensus tree with bootstrap support values obtained from analysis with 100 bootstrap replicates in GARLI 2.0.

From these 17 specimens, DNA was extracted from either entire worker adults or pupae using a DNeasy Tissue Kit (Qiagen Inc., Valencia, California, U.S.A.), following the manufacturer’s protocol but eluting the extract in sterilized water rather than the supplied buffer and at half the suggested volume. I used either a non-destructive method (cuticle of ant pierced prior to extraction, mostly used for adults), enabling me to retain and re-mount voucher specimens after extractions, or a destructive technique (entire ant pulverized, mostly used for pupae) in cases where multiple individuals from the same colony series were available. Three nuclear protein-coding genes were amplified:long wavelength rhodopsin (LW Rh, 856bp exon /255bp intron), arginine kinase (ArgK, 388bp exon/177bp intron) and carbamoylphosphate synthase (CAD, 529bp exon/252bp intron). The sequence lengths given here refer to the aligned sequence data included in phylogenetic inference and add up to a total of 2457bp. The three amplified genes are widely used for phylogenetic inference in ants and primers are available (Ward and Downie 2005; Brady et al. 2006; Moreau et al. 2006; Ward et al. 2010; Blaimer in prep.), and their usefulness in phylogenetic inference between closely related species has been demonstrated (Lucky 2011; Blaimer 2012). Amplifications were performed using standard PCR methods outlined in Ward and Downie (2005) and sequencing reactions were analyzed on an ABI 3730 Capillary Electrophoresis Genetic Analyzer with ABI BigDye Terminator v3.1 Cycle Sequencing chemistry (Applied Biosystems Inc., Foster City, CA). All sequences have been deposited in GenBank, with accession numbers listed in Table 1; the data matrix and tree used to create Fig. 1 have further been deposited in TreeBase (ID 12240; available at: http://purl.org/phylo/treebase/phylows/study/TB2:S12240).

Sequence data were assembled and edited in the program SEQUENCHER 4.6 (Gene Codes Corporation, 2006, Ann Arbor, MI), aligned in CLUSTALX 2.0.12 (Thompson et al. 1997; Larkin et al. 2007), and corrected by eye in MACCLADE 4.08 (Maddison and Maddison 2000). Phylogenetic analyses within a Bayesian framework (BI hereafter) were performed using MRBAYES v3.1 (Ronquist and Huelsenbeck 2003), accessed through the CIPRES science gateway (Miller et al. 2010); analyses within a maximum likelihood framework (ML hereafter) used GARLI v2.0 (Zwickl 2006) and were performed on an IMac desktop computer. BI- and ML-analyses were based on a concatenated data matrix of the three loci. The data matrix was divided into nine data subsets by gene, translational pattern (exon, intron) and codon position (1^st^ + 2^nd^ vs 3^rd^). Best-fitting models of nucleotide sequence evolution were selected for each partition using the Akaike information criterion (AIC) in the program MRMODELTEST v2.3 (Posada and Crandall 1998; Nylander et al. 2004) for application in BI-analyses, and in MODELTEST v3.7 (Posada and Crandall, 1998) for specification in ML-analyses, both executed through PAUP* 4.0b10 (Swofford 2000). Selected models for each data subset can be found in Table 2.

**Table 2.** **Data partitions and selected substitution models.** Information on data subsets, including number of bases, number of variable characters (VC), number of parsimony-informative characters (PIC) and substitution models selected for the respective partition using the Akaike information criterion in MRMODELTEST v2.3 (Posada and Crandall 1998; Nylander 2004) or MODELTEST v3.7 (Posada and Crandall 1998) for application in BI- or ML-analyses respectively.

**Data partition**	**No. bases**	**No. VC**	**No. PIC**	**Substitution model - BI**	**Substitution model - ML**
LW Rh exons position 1 + 2	570	10	17	HKY+I	HKY+I
LW Rh exons position 3	286	34	17	HKY	K81uf
LW Rh introns	255	25	21	HKY	TrN
ArgK exons position 1 + 2	258	3	8	K80	K80
ArgK exons position 3	130	15	18	HKY	TrN
ArgK introns	177	11	15	HKY	K81uf
CAD exons positions 1 + 2	352	13	13	HKY	HKY
CAD exons positions 3	177	23	18	SYM+G	TVMef+G
CAD introns	252	34	11	HKY	TrN
entire dataset	2457				

BI-analyses each employed two runs of Metropolis-coupled Markov Chain Monte Carlo (MCMCMC) consisting of four chains (temp=0.05) and sampling every 1000 generations. The model parameters transition-transversion ratio, gamma shape, proportion of invariable sites, rate matrix and state frequencies were unlinked across partitions, and a variable rateprior was employed to allow for rate variation among partitions. Convergence of chains and other diagnostic values were assessed in several ways. In MRBAYES I confirmed that the ASDSF had reached values well below 0.01 and PSRF values had approached 1.0 for all parameters. In TRACER v1.5 (Rambaut and Drummond 2007), convergence was confirmed visually and mixing of chains was evaluated with effective sample size (ESS) values. To assess whether tree topologies were sampled in proportion to their true posterior distribution, I further used the compare, slide and cumulative plotting functions on the AWTY-online server (Wilgenbusch et al. 2004). All the above indicators returned good values after MCMCMC-sampling for 20 million generations; consensus trees were summarized in MRBAYES after discarding 25% of samples as burnin. I further performed a ML-search for the best scoring tree (results not shown), as well as a bootstrap search with 100 replicates in GARLI. Program configuration settings were left at defaults. Trees resulting from the bootstrap search were summarized as majority-rule consensus tree in PAUP* 4.0b10 (Swofford 2000).

Ancillary genetic data supporting the results outlined below has been generated through the joint barcoding initiative of Malagasy ants by the California Academy of Sciences and the Biodiversity Institute of Ontario, Guelph, Canada (www.barcodinglife.org). The barcoding region of cytochrome oxidase I (COI) for ~130 specimens of five of the six below recognized species (with variable taxon sampling of 2–101 individuals per species) was thus available to guide taxonomic decisions. Analyses of these data are to be published elsewhere.

## Results

### Molecular results

All molecular phylogenetic analyses (BI and ML) of the data strongly suggest that there are six species of Malagasy *Orthocrema*, namely the previously described *Crematogaster rasoherinae* and *Crematogaster madecassa* and four new species: *Crematogaster telolafy* sp. n., *Crematogaster razana* sp. n., *Crematogaster volamena* sp. n. and *Crematogaster mpanjono* sp. n. (Figure 1). The previously described *Crematogaster voeltzkowi* from the Comoros Islands shows little genetic differentiation from *Crematogaster rasoherinae*, warranting synonymy with the latter (as indicated in Figure 1A). The same applies to *Crematogaster rasoherinae brunneola* (as indicated in Figure 1A). Further supported is the presence of two distinct species-groups, the *Crematogaster madecassa*-group and the *Crematogaster volamena*-group, with members as listed below. *Crematogaster rasoherinae* is shown as quite distantly related to the *Crematogaster madecassa* and *Crematogaster volamena* species-groups, which in turn also clearly do not form a monophyletic grouping. This suggests these two species groups and *Crematogaster rasoherinae* have originated from separate ancestors and three colonizations of the Malagasy region took place within the *Orthocrema* lineage. The exact relationships of these two species-groups and of *Crematogaster rasoherinae* to each other, and to the non-Malagasy taxa *Crematogaster arcuata* and *Crematogaster* cf. *dolens* remain unclear as they receive only moderate support in the BI analysis (Figure 1A), and are unresolved in the ML analysis (Figure 1B).

### Species list and species-groups of the subgenus *Orthocrema* in the Malagasy region

***Crematogaster rasoherinae* Forel, 1891**

= *Crematogaster rasoherinae* var. *brunneola* Emery, 1922 (replacement name for *Crematogaster rasoherinae* var. *brunnea* Forel, 1907),**syn. n.**

= *Crematogaster voeltzkowi* Forel, 1907, **syn. n.**

***Crematogaster madecassa*-group:**

*Crematogaster madecassa* Emery, 1895

*Crematogaster telolafy* **sp. n.**

*Crematogaster razana* **sp. n.**

***Crematogaster volamena*-group:**

*Crematogaster volamena* **sp. n.**

*Crematogaster mpanjono* **sp. n.**

### Diagnosis of the subgenus *Orthocrema* in the Malagasy region

**Workers**

**Figures 2–5.**
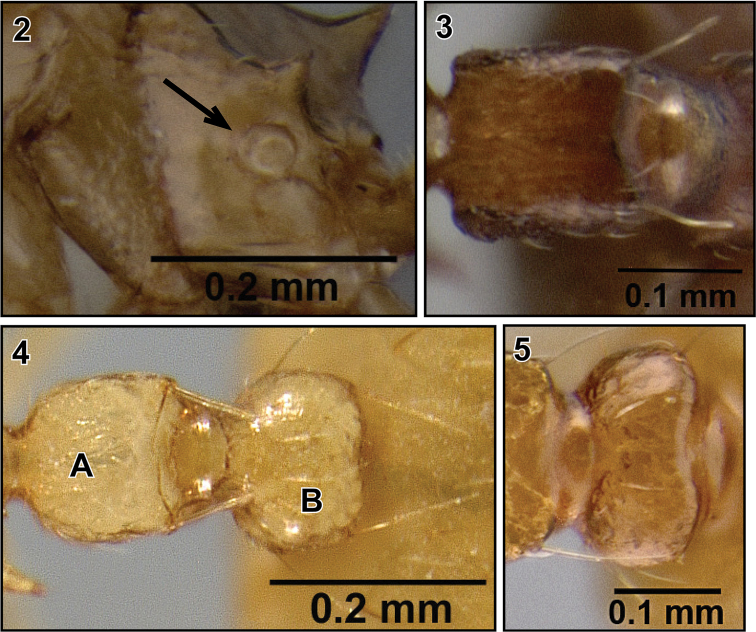
Worker diagnoses of Malagasy *Crematogaster* (*Orthocrema*). **2** propodeal spiracle circular (*Crematogaster razana*, CASENT0149655) **3** petiole in dorsal view rectangular (*Crematogaster rasoherinae*, CASENT0070841) **4 A** petiole in dorsal view ovo-rectangular **4** **B** postpetiole globular (*Crematogaster telolafy*, CASENT0419808) **5** postpetiole with broad impression (*Crematogaster volamena*, CASENT0077219).

1. Very small to medium-sized (HW 0.43–0.98, WL 0.44–0.95).

2. Antennae 11-segmented, antennal club 2-segmented.

3. Promesonotal suture absent.

4. Lateral margins of promesonotum with at least 4 long, erect setae.

5. Propodeal spiracle circular or subcircular (Figure 2).

6. Petiole in dorsal view rectangular (Figure 3) or ovo-rectangular (Figure 4A).

7. Petiole with dorsoposterior lateral denticles or tubercules that each bear an erect seta (Figures 3 and 4A).

8. Postpetiole either more or less globular (Figure 4B), without median longitudinal impression, or weakly bilobed with a broad impression (Figure 5).

9. Postpetiole with at least one pair of long, dorsoposterior setae (Figure 4B).

10. Subpetiolar process present (form variable).

11. Sculpture overall reduced, mostly aciculate, small regions areolate or reticulate.

**Minimal diagnosis**

A combination of characters 6, 7, 8 and 9 will unequivocally separateworkers of *Orthocrema* species from the remaining *Crematogaster* species in the Malagasy region.

**Queens**

**Figures 6–11.**
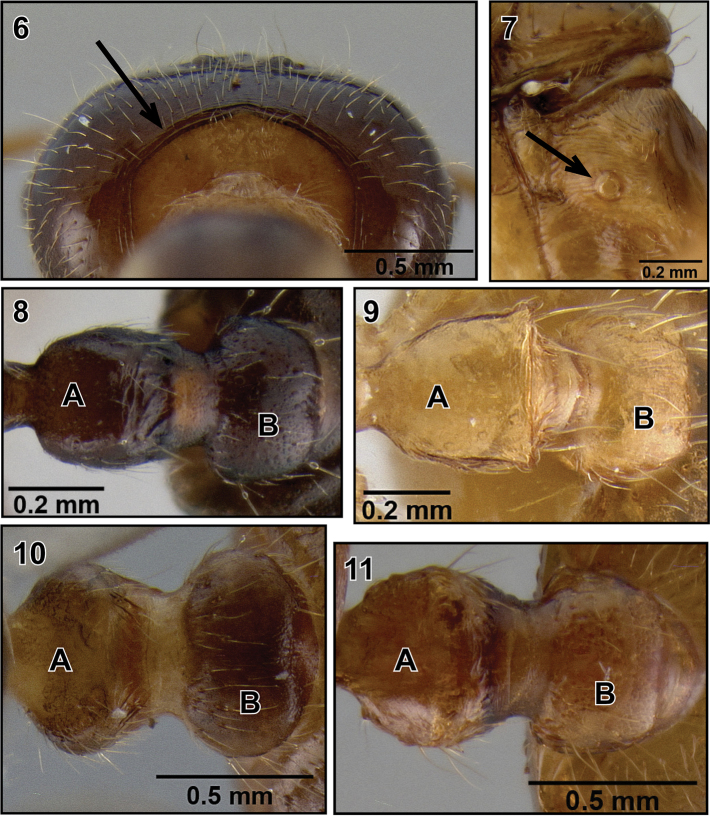
Queen diagnoses of Malagasy *Crematogaster* (*Orthocrema*). **6** occipital carinae distinct (*Crematogaster volamena*, CASENT0161415) **7** propodeal spiracle circular (*Crematogaster razana*, CASENT0148782) **8** **A** petiole rectangular **B** postpetiole globular (*Crematogaster rasoherinae*, CASENT0193403) **9 A** petiole ovo-rectangular **B** postpetiole globular (*Crematogaster madecassa*, CASENT0436253); **10 A** petiole oval **B** postpetiole globular (*Crematogaster volamena*, CASENT0161415) **11 A** petiole subquadrate **B** postpetiole globular (*Crematogaster mpanjono*, CASENT0067033).

1. Very small to large (HW 0.73–1.72, WL 0.83–2.70).

2. Antennae 11-segmented, antennal club weakly 2-segmented.

3. Occipital carinae mostly present (Figure 6).

4. ropodeal spiracle circular (Figure 7) or subcircular.

5. Petiole in dorsal view rectangular (Figure 8A), ovo-rectangular (Figure 9A), oval (Figure 10A) or subquadrate (Figure 11A).

6. Postpetiole more or less globular, without distinct median longitudinal impression (Figures 8–11B).

**Minimal diagnosis**

A combination of characters 3, 5 and 6 will unequivocally separatequeens of *Orthocrema* species from the remaining *Crematogaster* species in the Malagasy region.

### Key to the workers of *Crematogaster* (*Orthocrema*) species in the Malagasy region

**Table:** 

1	Petiole in dorsal view rectangular (Figure 3), with both antero- and posterolateral denticles; abdominal tergite 4 with sparse erect pilosity, often only a single row of setae towards posterior end	*Crematogaster rasoherinae*
–	Petiole in dorsal view ovo-rectangular (Figure 4A), with only posterolateral denticles present; abdominal tergite 4 with abundant erect pilosity throughout	2
2(1)	Occipital carinae distinct and sharp (Figure 12); eyes larger (OI 0.22–0.28) and distinctly protruding (as in Figure 27A and 29A); propodeum with raised, sharp lateral carinae, confluent with propodeal spines (Figure 13)	3
–	Occipital carinae indistinct (Figure 14); eyes smaller (OI 0.18–0.22) and less protruding (as in Figure 31A and 33A); propodeum lacking raised, sharp lateral carinae (Figure 15)	5
3(2)	One pair of long, flexuous setae present on posterior end of lateral mesonotal carinae; clypeus with two distinct median vertical carinae (Figure 16); antennal scapes reaching, or well surpassing posterior margin of head (SI 0.78–1.01); subpostpetiolar process usually present	4
–	Long, flexuous setae absent from posterior end of lateral mesonotal carinae; clypeus lacking median vertical carinae (Figure 17); antennal scapes shorter, barely reaching head margin (SI 0.74–0.77); subpostpetiolar process absent	*Crematogaster razana*
4(3)	Antennal scapes well surpassing posterior margin of head (SI 0.85–1.01); propodeal spines medium-sized (SPI 0.17–0.26), usually thin and acute (Figure 18A), in lateral view directed upwards but straight	*Crematogaster madecassa*
–	Antennal scapes just reaching posterior margin of head (SI 0.78–0.87); propodeal spines shorter (SPI 0.10–0.19), usually in form of acute triangular points (Figure 18B), if more elongate and spiniform, then distinctly curved upwards (Figure 18C)	*Crematogaster telolafy*
5(2)	Propodeal spines shorter (SPI 0.06–0.09); propodeum often with longer erect pilosity; *rare, Madagascar: Nosy Bé, R.S. Manongarivo, Ile St. Marie*	*Crematogaster mpanjono*
–	Propodeal spines often longer (SPI 0.06–0.12); propodeum lacking longer erect pilosity; *more common*, *eastern rainforests of Madagascar*	*Crematogaster volamena*

Note: *Crematogaster volamena* and *Crematogaster mpanjono* can only be reliably identified based on queen characters and genetic data.

**Figures 12–18.**
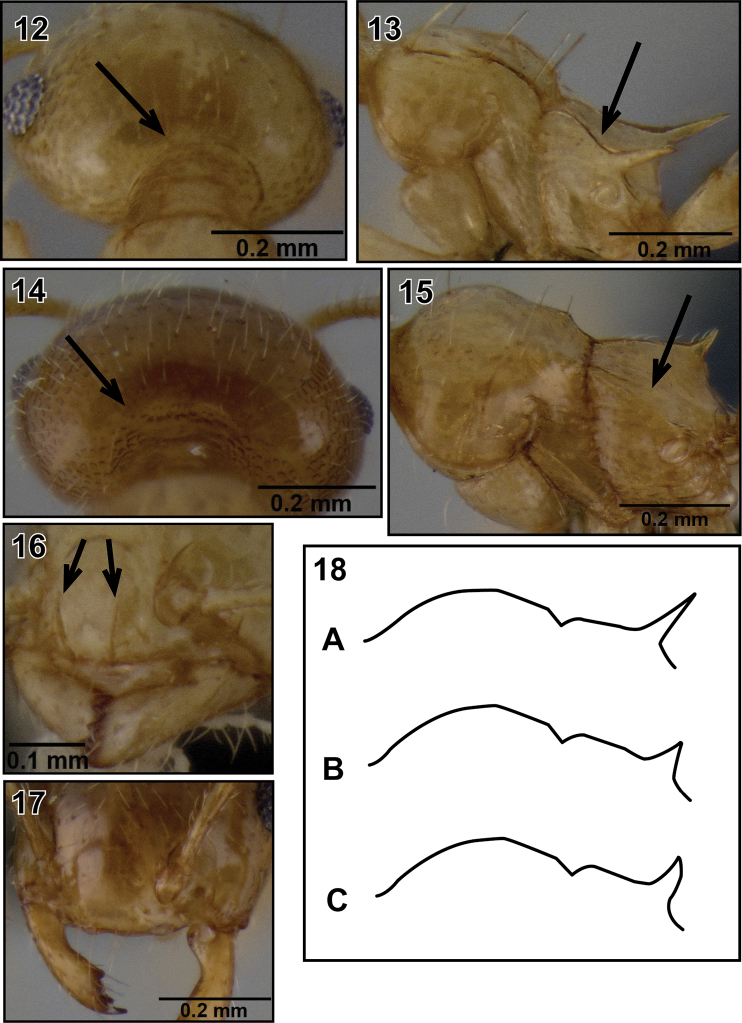
Species key to the workers of Malagasy *Crematogaster* (*Orthocrema*). **12** occipital carinae distinct (*Crematogaster madecassa*, CASENT0038498) **13** propodeum with lateral raised carinae (*Crematogaster madecassa*, CASENT0038498) **14** occipital carinae indistinct (*Crematogaster volamena*, CASENT0125748) **15** propodeum without lateral raised carinae (*Crematogaster volamena*, CASENT0125748) **16** clypeus with two median carinae (*Crematogaster madecassa*, CASENT0038498) **17** clypeus without median carinae (*Crematogaster razana*, CASENT1408782) **18 A**  propodeal spines thin and acute **B** propodeal spines triangular **C** propodeal spines curved-triangular.

### Key to the queens of *Crematogaster* (*Orthocrema*) species in the Malagasy region (except *Crematogaster telolafy* which is unknown)

**Table:** 

1	Propodeal spines present	2
–	Propodeal spines absent	3
2(1)	Body size smaller (HW 0.89–1.03,WL 1.28–1.53); propodeal spines longer (SPI 0.04–0.14); clypeus lacking median notch (Figure 19); antennal scapes usually surpassing posterior margin of head	*Crematogaster madecassa*
–	Body size larger (HW 1.10, WL 1.74); propodeal spines reduced to minute dents (SPI 0.02); clypeus with a median notch (Figure 20); antennal scapes just reaching posterior margin of head	*Crematogaster razana*
3(2)	Body size very small (HW 0.80–0.89, WL 1.50–1.63); eyes large (OI 0.30–0.34)	*Crematogaster rasoherinae*
–	Body size large (HW 1.48–1.72, WL 2.61–2.70); eyes medium-sized (OI 0.23–0.27)	4
4(3)	Head wider than long (CI 1.08); occipital carinae well pronounced (Figure 22); scuto-scutellar suture broadly meeting mesoscutum (Figure 21); dorsal face of propodeum short	*Crematogaster volamena*
–	Head longer than wide (CI 0.96); occipital carinae indistinct; scuto-scutellar suture acutely meeting mesoscutum (Figure 23); dorsal face of propodeum about as long as posterior face	*Crematogaster mpanjono*

**Figures 19–23.**
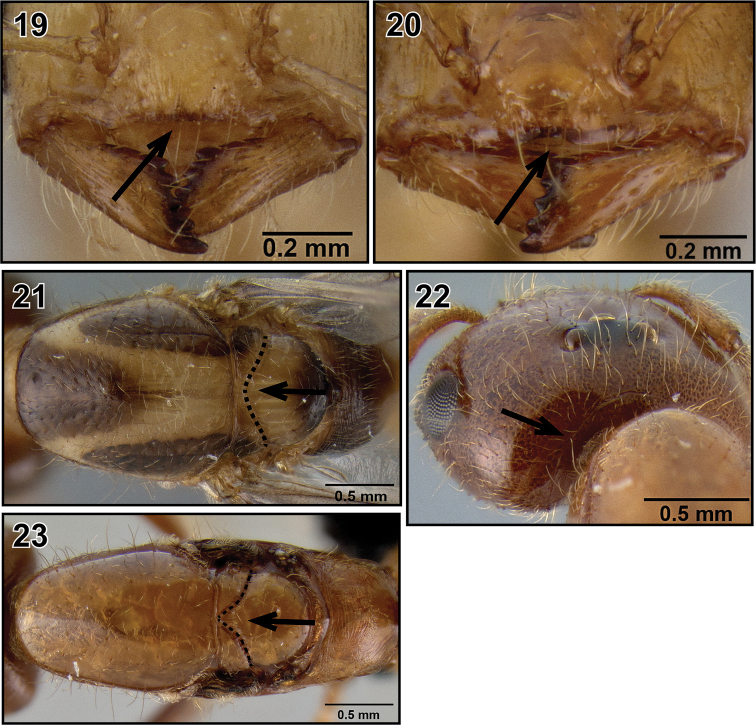
Species key to the queens of Malagasy *Crematogaster* (*Orthocrema*). **19** clypeus without median notch (*Crematogaster madecassa*, CASENT0040391) **20** clypeus with median notch (*Crematogaster razana*, CASENT0148782) **21** scuto-scutellar suture broadly meeting mesoscutum (*Crematogaster volamena*, CASENT0161415) **22** occipital carinae distinct (*Crematogaster volamena*, CASENT0161415) **23** scuto-scutellar suture acutely meeting mesoscutum (*Crematogaster mpanjono*, CASENT0067033).

#### Crematogaster rasoherinae

Forel

http://species-id.net/wiki/Crematogaster_rasoherinae

**Figure 24.**
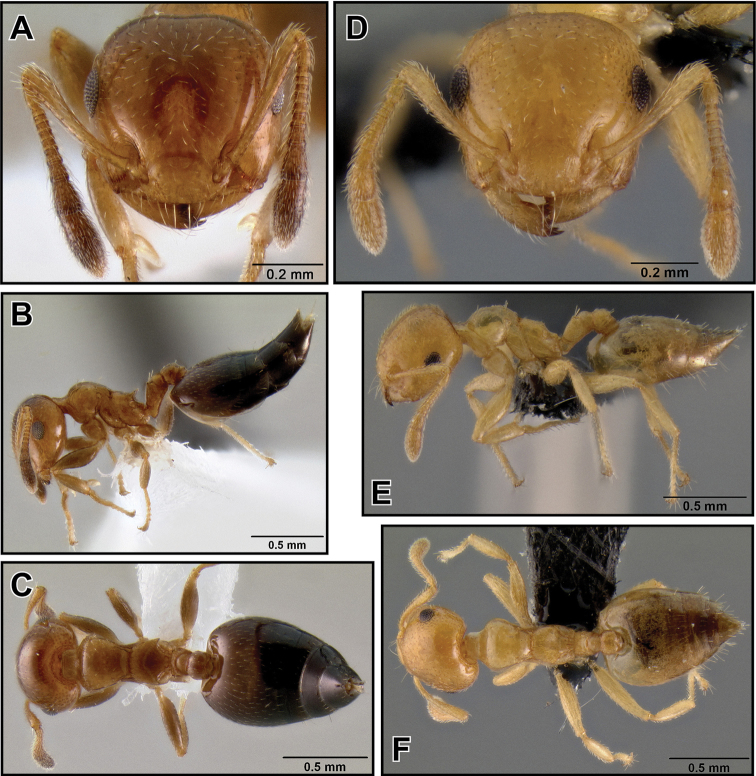
*Crematogaster rasoherinae*, workers. **A–C** form with propodeal spines (CASENT0193412) **A** full face **B** lateral **C** dorsal **D–F** form without propodeal spines (CASENT0147430) **D** full face **E** lateral **F** dorsal.

Figures 24 [Fig F7] 26

Crematogaster rasoherinae Forel, 1891: 194. Worker syntype(s) from MADAGASCAR: Tamatave (O’swald) [**Naturhist. Mus. Hamburg, not examined, destroyed during WWII**]. Forel, 1912: 164. Queen, male described. Combination in *Crematogaster* (*Neocrema*): Santschi, 1918: 182; in *Crematogaster* (*Crematogaster*): Wheeler, W.M. 1922: 1023; Combination in *Crematogaster* (*Orthocrema*): Emery, 1922: 131; Combination in *Crematogaster* (*Mesocrema*): Santschi, 1928: 33.
= Crematogaster (*Orthocrema) rasoherinae* var. *brunneola* Emery, 1922: 131. Replacement name for *Crematogaster rasoherinae* var. *brunnea* Forel, 1907: 79. Worker and queen syntypes from Madagascar: Andranohinaly (SW Madagaskar) (Voeltzkow) [**MHNG, examined**]. [Junior primary homonym of *brunnea* Smith, F. 1857: 75.]. **Lectotype worker** by present designation: top specimen of 2 workers on one pin, CASENT0101836 (image on AntWeb). Combination in *Crematogaster* (*Mesocrema*): Santschi, 1928: 33. **Syn. n.**
= Crematogaster voeltzkowi Forel, 1907: 78. Worker syntypes from Comoros: Anjouan (Voeltzkow) [**MHNG, examined**]. **Lectotype worker** by present designation: top specimen of 2 workers on one pin, CASENT0101615 (image on AntWeb). Combination in *Crematogaster* (*Neocrema*): Santschi, 1918: 182 (misspelled as *woelzkowi*); Comb. in *Crematogaster* (*Crematogaster*): Wheeler, W.M. 1922: 1024; Comb. in *Crematogaster* (*Orthocrema*): Emery, 1922: 131. **Syn. n.**

##### Type material.

Syntypes not available for examination; these specimens were housed in the collection of the Naturhistorisches Museum in Hamburg and were destroyed during World War II (confirmation obtained 19.vii.2011, via e-mail communication with F. Wieland).

**Neotype** worker, by present designation: pinned, CASENT0120911, BLF16755, ex dead twig above ground; original locality label: Prov. Toamasina, Tamatave, 20m, 18°09.28'S, 49°24.76'E, 16.ii.2007, urban gardens, Fisher et al. BLF16755; deposited at CASC.

##### Other material examined

(BBBC, CASC, MHNG, NHMB, PSWC, ZMBH, MCZC). MADAGASCAR: *Antsiranana*: Baie Sakalava: -12.27330, 49.39064, 10m (B.L.Fisher et al.); Montaigne Français: -12.32278, 49.33817, 180m (R.Harin’Hala); 7 km N Joffreville: -12.33333, 49.25000, 360m (R.Harin’Hala); 2km S Joffreville: -12.47639, 49.22222, 500m (G.Alpert); 7km SE Antsiranana: -12.31670, 49.33330, 80m (G.Alpert); R.S. Ambre:-12.46889, 49.24217, 325m (B.L.Fisher et al.); P.N. Montagne d’Ambre: -12.50035, 49.17500, 885m; -12.53444, 49.17950, 925m; -12.52028, 49.17917, 1125m (B.L.Fisher et al.); Nosy Bé, R.N.I. Lokobé: -13.41944, 48.33117, 30m (B.L.Fisher et al.); Nosy Bé, Lokobe Forest: -13.41640, 48.30720, 50m (G.Alpert); Nosy Bé, 5km E Marodokana: -13.36670, 48.30000, 50m (G.Alpert); R.S. Manongarivo: -13.93153, 48.45213, 370m (B.B.Blaimer); Ambondrobe: -13.71533, 50.10167, 10m (B.L.Fisher et al.); P.N. Ankarana: -12.90889, 49.10983, 80m; -12.86361, 49.22583, 210m (B.L.Fisher et al.), P.N. Ankarana: -12.90056, 49.14722, 150m (G.Alpert); F Andavakoera: -13.11833, 49.23000, 425m (B.L.Fisher et al.); Rés. Analamerana: -12.74667, 49.49483, 60m (B.L.Fisher et al.); F Binara: -13.26333, 49.60333, 650–800m (B.L.Fisher et al.); 6.3 km S Ambanizana: -15.68131, 49.9580, 25m (B.L.Fisher et al.); 5.3 km SSE Ambanizana, 425m, -15.66667, 49.96667 (B.L.Fisher et al.); Nosy Mangabe: -15.49730, 49.76223, 5m (B.L.Fisher et al.); -15.50000, 49.76670, 200m (P.S.Ward); P.N. Marojejy: -14.43333, 49.78333, 450m (B.L.Fisher et al.); P.N. Masoala: -15.71333, 49.97167 (B.L.Fisher et al.); -15.72667, 49.95667, 150m (A.Dejean et al.); 84km SW Sambava on road to Andapa: -14.57730, 49.73940, 160m (W.L.&D.E.Brown); Vohemar: -13.35967, 50.00390, 16m (B.L.Fisher et al.); F Analabe: -13.08333, 49.90833, 30m (B.L.Fisher et al.); Forêt d’Ampondrabe: -12.97000, 49.70000, 175m (B.L.Fisher et al.); F Orangea: -12.25889, 49.37467, 90m (B.L.Fisher et al.);F Ampombofofo: -12.09949, 49.33874, 25m (B.L.Fisher et al.); Ampamakiambato: -13.97545, 48.15929, 145m (B.L.Fisher et al.); Forêt d’Anabohazo: -14.30889, 47.91433, 120m (B.L.Fisher et al.); Ankobahoba: -13.39166, 48.48249, 40m (B.L.Fisher et al.); 14km W Cap Est, Ambato: -15.29128, 50.33803, 150m (B.L.Fisher et al.); Andranomatàna: -13.14965, 48.91765, 28m (B.L.Fisher et al.); Tsihombe: -25.31833, 45.48367, 30m (B.L.Fisher et al.); Antalaha: -14.90130, 50.28095, 24m (B.L.Fisher et al.); 55km S Antalaha, Nosy Ngontsy: -15.26440, 50.48930, 50m (G.Alpert); 55km S Antalaha, Cap Est: -15.25640, 50.47940, 1m (G.Alpert); Ambohitsara, 10km SW Antalaha: -14.95000, 50.26670, 50m (G.Alpert); *Antananarivo*: R.S. Ambohitantely: -18.19800, 47.28150, 700m (B.L.Fisher et al.); *Fianarantsoa*: P.N. Ranomafana: -21.26650, 47.42017, 1020m (B.L.Fisher et al.); 3km W Ranomafana, nr Ifanadiana: -21.25000, 47.41670, 950m (P.S.Ward); Ranomafana, nr. Ifanadiana: -21.26670, 47.45000, 650m (P.S.Ward); 10km E Ranomafana: -18.99972, 48.95000, 50m (G.Alpert); R.S. Manombo: -23.01580, 47.71900, 30m (B.L.Fisher et al.); -23.02183, 47.72000, 36m (R.Harin’Hala); Mahabo [Rés. Forestière d’Agnalazaha]: -23.19383, 47.72300, 20m (B.L.Fisher et al.); F Ampitavananima: -23.12972, 47.71700, 34m (B.L.Fisher et al.); 8km E Kianjavato: -21.38860, 47.94360, 145m (G.Alpert); *Mahajanga*: PN Ankarafantsika (F Tsimaloto): -16.22806, 47.14361, 135m (B.L.Fisher et al.); PN Ankarafantsika: -16.31670, 46.81670 (L.A. Nilsson); Ambolomaiky: -15.85410, 46.74663, ca. 80m (B.L.Fisher et al.); Forêt Ambohimanga: -15.96267, 47.43817, 250m (B.L.Fisher et al.); PN Baie de Baly: -16.01000, 45.26500, 10m (B.L.Fisher et al.); P.N. Namoroka: -16.37667, 45.32667, 100m (B.L.Fisher et al.); P.N. Tsingy de Bemaraha: -19.13222, 44.81467,100m; -18.70944, 44.71817, 150m (B.L.Fisher et al.); Mahavavy River: -16.05167, 45.90833, 20m (B.L.Fisher et al.); Rés. Forestière Beanka: -18.02649, 44.05051, 250m (B.L.Fisher et al.); F Tsimembo: -19.02139, 44.44067, 20m; -18.99528, 44.44350, 50m (B.L.Fisher et al.); S.F. Ampijoroa: -16.31944, 46.81333, ca. 40m; -16.31670, 46.81670, 80m; F Asondrodava: : -17.96533, 44.03550, 6m (R.Harin’Hala); 3km S Namakia: -15.95611, 45.83556, 40m (G. Alpert); *Toamasina*: RS Ambatovaky: -16.81739, 49.29402, 360m (B.L.Fisher et al); F Ambatovy: -18.85083, 48.32000, 1075m (B.L.Fisher et al.); Rés. Betampona: -17.92400, 49.19967, 390m (B.L.Fisher et al.); 11km SE Ampasimanolotra (=Brickaville): -18.90000, 49.13330, 5m (P.S.Ward); 10km N Brickaville: -18.79194, 49.08667, 100m(G. Alpert); F Kalalao [Ile St.Marie]: -16.92250, 49.88733, 100m (B.L.Fisher et al.); F Ambohidena [Ile St.Marie]: -16.82433, 49.96417, 20m (B.L.Fisher et al.); F Ampanihy [Ile St.Marie]: -16.91117, 49.93917, 10m (B.L.Fisher et al.)**;** F Sahafina: -18.81445, 48.96205, 100m; -18.81445, 48.96205, 140m (B.L.Fisher et al.); Mahavelona (Foulpointe): -17.66667, 49.50000, (A.Pauly); Manankinany: -17.03330, 49.53330 (L.A.Nilsson); Tanambao Nosibe: -17.89117, 49.45617, 15m (Blaimer&Raharimalala); Antaratasy: -17.76733, 49.47767, 25m (Blaimer & Raharimalala); Ampasina-Maningory: -17.21467, 49.40550, 20m (Blaimer & Raharimalala); Anosintany: -16.91117, 49.58867, 10m (Blaimer & Raharimalala); Maitsokely: -16.90617, 49.58683, 10m (Blaimer & Raharimalala); Fenoarivo: -17.38117, 49.41500, 10m (Blaimer & Raharimalala); Antetezambaro: -17.05283, 49.56700, 10m (Blaimer & Raharimalala); Mahambo: -17.48933, 49.45167, 10m (Blaimer & Raharimalala); Tamatave: -18.15467, 49.41267, 20m (B.L.Fisher et al.); Brickaville:-18.82183, 49.07017, ca. 25m (B.L.Fisher et al.); Analalava: -17.7095, 49.45400, 50m (B.L.Fisher et al.); Mahanoro: -19.89933, 48.80883, 15m (B.L.Fisher et al.); Vatomandry: -19.33283, 48.97950, 16m (B.L.Fisher et al.); Forêt d’Analava Mandrisy: -16.48567, 49.84700, 10m (B.L.Fisher et al.); S.F. Tampolo: -17.28250, 49.43000, 10m (B.L.Fisher et al.); Analalava: -17.693194, 49.46027, ca. 20m (R.Harin’Hala); *Toliara*: Mahafaly Plateau: -24.65361, 43.99667, 80m (B.L.Fisher et al.); F Mikea: -22.90367, 43.47550, 35m (R. Harin’Hala); Libanona Beach: -25.03883, 46.99600, 20m (B.L.Fisher et al.); F Petriky: -25.06167, 46.87000, 10m (B.L.Fisher); Ranobe: -23.03975, 43.61090, 30m (Frontier Project, MGF); Rés. Berenty (F Bealoka): -24.95694, 46.27150, 35m (B.L.Fisher et al.); Rés. Berenty (F Malaza): -25.00778, 46.30600, 40m; Rés. Berenty (F Anjapolo): -24.92972, 46.20967, 65m (B.L.Fisher et al.); Rés. Berenty: -25.02100, 46.30550, 35m, -25.00670, 46.30330, 85m (R.Harin’Hala), -25.01670, 46.30000, 35m (P.S.Ward), -24.98330, 46.30000, 30m; Miandrivazo: -19.52317, 45.4575, 80m (B.L.Fisher et al.); Morondava: -20.29650, 44.28150, ca. 10m (B.L.Fisher et al.); F Beroboka: -22.23306, 43.36633, 80m (B.L.Fisher et al.); F Tsinjoriaky: -22.80222, 43.42067, 70m (B.L.Fisher et al.); PN Tsimanampetsotsa: -24.10056, 43.76000, 25m; -24.04722, 43.75317, 40m (B.L.Fisher et al.); Ejeda: -24.3505, 44.51600, 250m (B.L.Fisher et al.); F Tsivory: -24.07083, 46.07533, 400m (B.L.Fisher et al.); Manatantely:-24.9815, 46.92567, 100m (B.L.Fisher et al.); 6.1 km 182°S Marovato: -25.58167, 45.29500, 20m (B.L.Fisher et al.); 3.4 km 190° S Marovato: -25.55972, 45.28250, 160m (B.L.Fisher et al.); 3.5 km 236° SW Marovato: -25.55389, 45.25583, 230m (B.L.Fisher et al.); P.N. Andohahela: -24.81694, 46.61000, 150m (R.Harin’Hala); -24.93683, 46.62667, 180m (R.Harin’Hala); -24.75850, 46.85370, 275m; -24.93000, 46.64550, 300m (B.L.Fisher et al.); P.N. Andohahela/parcel3: -25.01366, 46.64650, 160m; -25.01790, 46.65175, 170m; P.N. Andohahela/ parcel1: -24.94713, 46.67312, 400m; -24.94683, 46.67625, 440m (B.B.Blaimer); 5km NNW Isaka-Ivondro, Rés. Andohahela: -24.75000, 46.85000, 280m (P.S.Ward); (P.S.Ward); 7km NW Ranopiso: -25.01670, 46.63330, 100m (P.S.Ward); 2.7km WNW 302º St.Luce: -24.77167, 47.17167, 20m (B.L.Fisher et al.); F Mandena: -24.95167, 47.00167, 20m (B.L.Fisher); Rés. Cap St.Marie: -25.58767, 45.16300, ca. 35m; -25.59444, 45.14683, 160m, -25.58167, 45.16833, 200m; (B.L.Fisher et al.); SW Madagaskar, Andranohinaly: -23.27500, 43.97500 (Voeltzkow).

SEYCHELLES: Mahé Island: Morne Blanc: -4.65988, 55.43743, 480m; -4.65740, 55.43325, 660m (B.L.Fisher et al.); Petite Congo Rouge: -4.64514, 55.43364, 745m (B.L.Fisher et al.); Mont Copolia : -4.65121, 55.45835, 520m (B.L.Fisher et al.); Silhouette Island: Mont Dauban: -4.48126, 55.22641; 735m (B.L.Fisher et al.); Aldabra Atoll: Grande Terre Isl.: -9.43453, 46.45767, ca. 5m (S.M.Goodman); COMOROS: Mohéli: Ouallah: -12.30668, 43.66407, 275m; -12.30353, 43.66827, 500m; -12.29696, 43.67392, 680m; -12.29600, 43.67600, 750m (B.L.Fisher et al.); Madahali: -12.37421, 43.86857, 50m (B.L.Fisher et al.); Lac Boundouni: -12.37915, 43.85165, 25m (B.L.Fisher et al.); Grande Comore: Goudjoulachamle: -11.44826, 43.27373, 80m (B.L.Fisher et al.); Grillé: -11.47578, 43.34669, 805m; -11.47578, 43.34669, 995m (B.L.Fisher et al.); Karthala: -11.82699, 43.42950, 1000m (B.L.Fisher et al.); Domani: -11.51778, 43.28000, 5m (B.L.Fisher et al.); Itoundzou: -11.63136, 43.30434, 635m (B.L.Fisher et al.); Trou du Prophete: -11.38087, 43.31335, 10m (B.L.Fisher et al.); Anjouan: -12.22265, 44.28820, 10m; -12.25764, 44.38915, 20m; -12.18771, 44.35929, 65m; -12.29311, 44.51090, 440m; -12.30537, 44.45031, 500m (B.L.Fisher et al.); Anjouan (Voeltzkow); Hajoho: -12.12195, 44.48795, 10m (B.L.Fisher et al.); Lac Dzialandée: -12.22474, 44.43121, 900m (B.L.Fisher et al.); Mt. Ntringui: -12.19865, 44.41866, 740m; -12.22043, 44.42924, 1225m (B.L.Fisher et al.); MAYOTTE: Reserve forestière Majimbini: -12.76796, 45.18615, 525m (B.L.Fisher et al.); Mont Combani: -12.80632, 45.15314, 370m (B.L.Fisher et al.); Baie de Tsingoni: -12.79260, 45.10764, 5m (B.L.Fisher et al.); Hajangoua: -12.85492, 45.19889, 10m (B.L.Fisher et al.); Mont Benara: -12.87585, 45.15672, 425m (B.L.Fisher et al.); Sazile: -12.97839, 45.17261, 35m (B.L.Fisher et al.); Tanaraki: -12.75754, 45.0678, 10m (B.L.Fisher et al.); Reserve forestiere Sohoa: -12.80586, 45.10054, 20m (B.L.Fisher et al.); Gorgora Kandza: -12.86735, 45.20827, 65m (B.L.Fisher et al.).

##### Worker measurements

**(n=28). Neotype worker:** HW 0.64; HL 0.61; EL 0.15; SL 0.45; WL 0.68; SPL 0.07; PTH 0.17; PTL 0.25; PTW 0.18; PPL 0.14; PPW 0.19; LHT 0.44; CI 1.05; OI 0.25; SI 0.74; SPI 0.10; PTHI 0.67; PTWI 0.73; PPI 1.41; LBI 1.53.

##### Other material.

HW 0.51–0.63; HL 0.49–0.62; EL 0.12–0.15; SL 0.38–0.46; WL 0.52–0.65; SPL 0.00–0.10; PTH 0.11–0.16; PTL 0.16–0.24; PTW 0.13–0.19; PPL 0.10–0.14; PPW 0.14–0.21; LHT 0.35–0.44; CI 0.99–1.07; OI 0.21–0.27; SI 0.71–0.82; SPI 0.00–0.17; PTHI 0.60–0.72; PTWI 0.65–0.93; PPI 1.21–1.62; LBI 1.26–1.56.

##### Diagnosis.

Workers of *Crematogaster rasoherinae* can be distinguished from all other Malagasy *Orthocrema* by the presence of small anterolateral denticles on the petiole and the rectangular shape of the same. Queens are distinct from all other species by their rectangular petiole shape. In addition, the absence of propodeal spines distinguishes *Crematogaster rasoherinae* queens from *Crematogaster madecassa* and *Crematogaster razana*, whereas very small size (HW 0.80–0.89, WL 1.50–1.63) and large eyes (OI 0.30–0.34) easily separate them from *Crematogaster volamena* and *Crematogaster mpanjono*.

##### Worker description

(Figures 24A–F).Very small species (HW 0.51–0.64, WL 0.52–0.68). Masticatory margin of mandibles with 4 teeth; clypeus with several weak vertical carinae; posterior margin of head in full face view usually laterally rounded, sometimes medially slightly depressed; occipital carinae well pronounced; antennal scapes usually just reaching, but not surpassing posterior margin of head; midline of eyes situated well above midline of head in full face view; eyes flush with head, not notably protruding.

Promesonotum laterally subangular, with mesonotum posterolaterally slightly marginate and metanotal groove bordered by weak carinae; in lateral view outline of promesonotum moderately convex; promesonotal suture usually absent; mesonotum with or without a distinct posterior face; metanotal groove with 2–3 median carinae of varying prominence; propodeal spines short (SPI < 0.17) or absent (most Comoros Isl. material), if present straight or upwards curved, in lateral view directed upwards, in dorsal view almost parallel and not diverging; dorsal face of propodeum very short; petiole in dorsal view rectangular, with dorsolateral margins weakly carinate or angular and small antero- and posterolateral denticles; subpetiolar process mostly developed as broad, rounded protuberance, sometimes as small angular dent; postpetiole more or less globular, merely impressed posteriorly, or with faint median impression; subpostpetiolar process often present as small, angular protrusion.

Head sculpture reduced, aciculate; mesosoma with promesonotum dorsally aciculate; meso- and metapleuron aciculate to areolate; propodeum with dorsal face carinulate or reticulate, posterior face shiny; dorsal face of petiole mostly reticulate; helcium dorsally finely areolate; postpetiole dorsally feebly reticulate; lateral and ventral face of petiole and postpetiole areolate or reticulate; face with 2–4 erect flexuous setae, and abundant short, subdecumbent pubescence; pronotum with 0–4 (most often 2) erect, stiff humeral setae, and 0–4 (usually 2) erect, stiff lateral setae on mesonotum, rarely also 2 erect setae present dorsally; mesosoma with scattered decumbent pubescence; petiole with a single stiff, erect seta on each posterolateral tubercle; postpetiole with a pair of erect dorsoposterior setae; abdominal tergites and sternites 4–7 with fairly abundant short erect pilosity (> 20 setae), which is more sparse on tergite 4 and usually present only towards posterior end, and with decumbent pubescence throughout.

Several color variants. Most widespread in Madagascar is a light to dark brown form; less common is a bicolored form with light brown or reddish head and mesosoma and dark gaster. On the Comoros islands, the Seychelles and Mayotte, *Crematogaster rasoherinae* is most often yellow or pale yellow colored, often with the posterior half of the gaster black. The typical brown Madagascar color form seems to be only present on the Seychelles.

##### Intermediate worker measurements

**(n=10).** HW 0.73–0.84, HL 0.72–0.83, EL 0.18–0.23, SL 0.49–0.56, WL 0.83–1.03, SPL 0.06–0.15, PTH 0.18–0.23, PTL 0.29–0.37, PTW 0.23–0.30, PPL 0.18–0.23, PPW 0.27–0.34 , LHT 0.50–0.57, CI 1.00–1.05, OI 0.24–0.30, SI 0.65–0.75, SPI 0.08–018, PTHI 0.56–0.67, PTWI 0.74–0.88, PPI 0.43–0.53, LBI 1.59–1.84.

##### Intermediate worker description

**Figure 25. F7:**
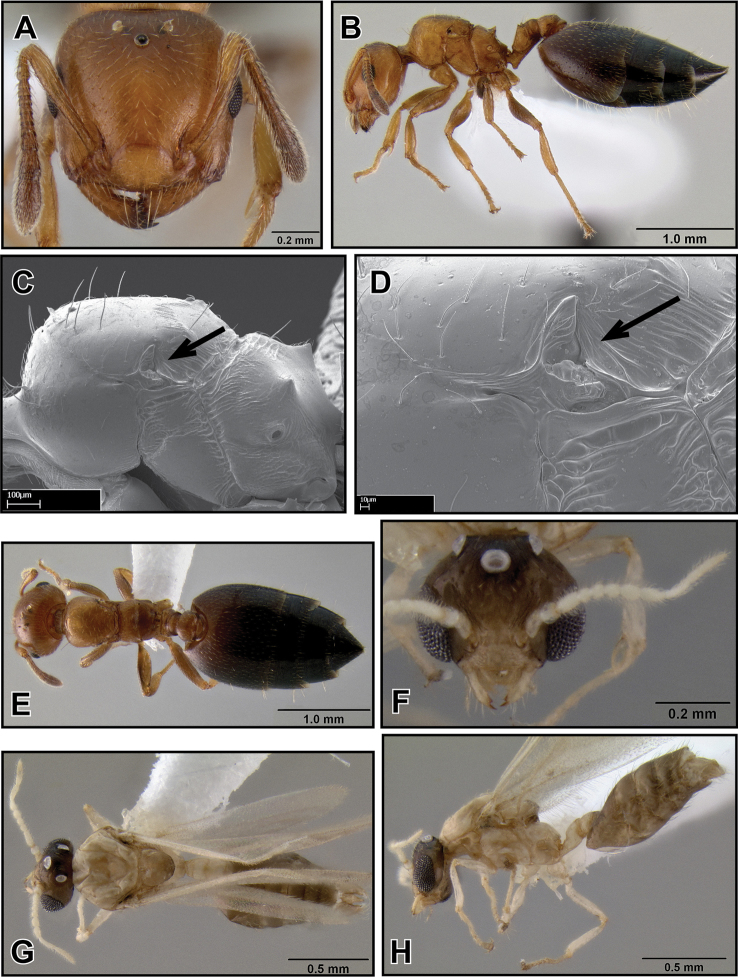
*Crematogaster rasoherinae*, intermorph and male. **A–E** intermorph(CASENT0193402) **A** full face **B** lateral **C** dorsolateral view of mesosoma (SEM) **D** close-up of mesosoma in dorsolateral view (SEM) **E** dorsal **F–G** male (CASENT0193414) **F** full face **G** dorsal **H** lateral.

(Figures 25A–E). Intermediate between workers and queens in size. Head, petiole and postpetiole characters similar to queens; ocelli present, but smaller than in queens; the mesonotum is to various extent raised and fused dorsally over pronotum and has wing attachment sutures; otherwise mesosomal characters more similar to worker characters, especially propodeum, and propodeal spines are present.

##### Queen measurements

**(n=10).** HW 0.80–0.89, HL 0.79–0.88, EL 0.26–0.28, SL 0.51–0.56, MSNW 0.62–0.85, MSNL 0.70–0.90, WL 1.50–1.63, SPL 0.00, PTH 0.20–0.24, PTL 0.35–0.43, PTW 0.26–0.31, PPL 0.21–0.28, PPW 0.31–0.37, LHT 0.62–0.70, CI 0.99–1.02, OI 0.30–0.34, SI 0.62–0.66, MSNI 1.72–1.91, SPI 0.13–0.16, PTHI 0.49–0.63, PTWI 0.62–0.75, PPI 1.30–1.63, LBI 2.19–2.48.

##### Queen description

**Figure 26.**
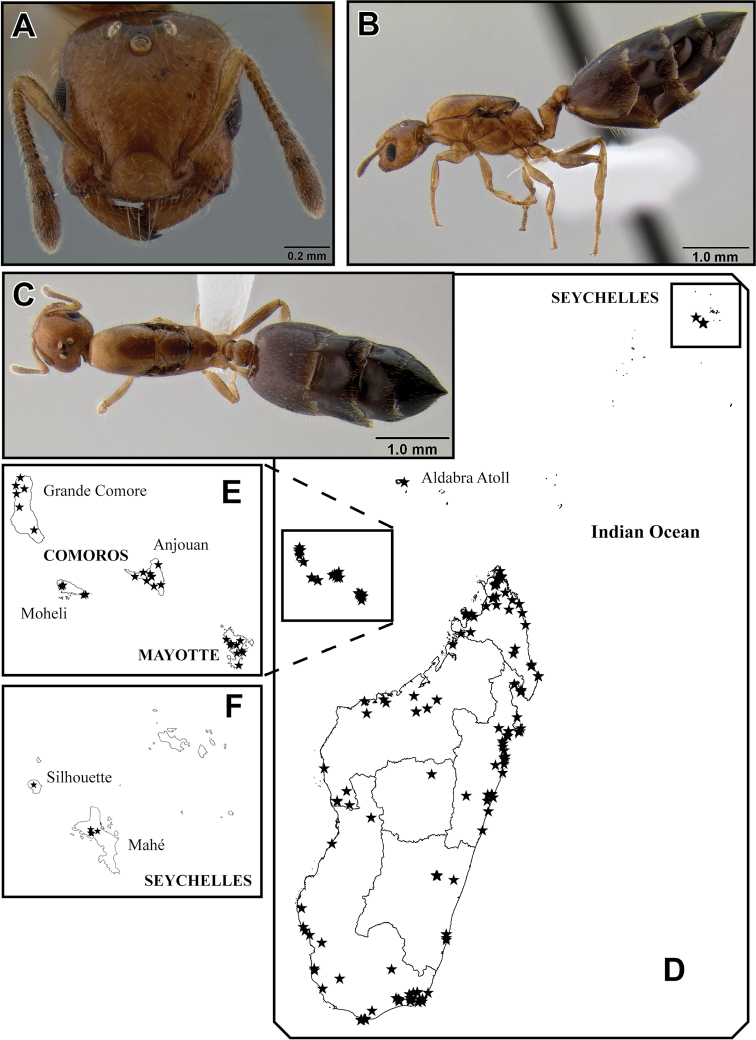
*Crematogaster rasoherinae*, queen and distribution. **A–C** queen (CASENT0193403) **A**  full face **B** lateral **C** dorsal **D–F** distribution maps **D** Madagascar and Indian Ocean island overview **E** Comoros and Mayotte **F** Seychelles.

(Figures 26 A–C). Very small (HW 0.80–0.89, WL 1.50–1.63). With worker characters, except as follows. Masticatory margin of mandibles with 5 teeth. Antennal scapes not surpassing posterior margin of head, reaching only to about level of lateral ocelli; eyes large (OI 0.30–0.34), situated at midline of head in full face view; head shape quadrate (CI 0.99–1.02), posterior margin of head straight.

Mesosoma slender (MSNI 1.72–1.91, WL 1.50–1.63); mesoscutum in dorsal view oval, about half as wide as long; dorsal face of propodeum distinct, about half as long as posterior face; propodeal spines absent; petiole and postpetiole as in worker; anteroventral subpetiolar tooth present, but reduced with respect to worker.

Sculpture smooth and shiny throughout; erect pilosity generally more abundant, but finer than in workers: face with 4–6 longer erect setae and abundant shorter erect to suberect pilosity; mesonotum with abundant short, and scattered longer erect setae; petiole with one pair of long flexuous setae posterior to posterior denticles; postpetiole with flexuous pair of dorsoposterior setae and 2–4 additional long setae; petiole and postpetiole with abundant shorter pilosity throughout. Body color similar to respective workers.

##### Male measurements

**(n=5).** HW 0.43–0.48, HL 0.34–0.37, EL 0.19–0.22, SL 0.05–0.08, MSNW 0.43–0.53, MSNL 0.38–0.49, WL 0.64–0.80, SPL 0.00, PTH 0.11–0.13, PTL 0.15–0.19 PTW 0.10–0.14, PPL 0.15–0.19, PPW 0.15–0.19, LHT 0.31–0.34, CI 1.19–1.33, OI 0.54–0.59, SI 0.15–0.21, MSNI 1.56–1.80, SPI 0.14–0.17, PTHI 0.64–0.74, PTWI 0.54–0.83, PPI 1.38–1.55, LBI 2.07–2.42.

##### Male description

(Figures 25F–H). Very small (HW 0.43–0.48, WL 0.64–0.80). Masticatory margin of mandibles with 2 teeth; eyes very large (OI 0.54–0.59) and protruding, covering most of gena, and reaching anteriorly almost to clypeal margin; antennae 9–10-segmented (separation between 3^rd^ and 4^th^ funicular segment often absent or incomplete), scapes very short (SI 0.15–0.21), 2^nd^ funicular segment globular, last 2 or 3 funicular segments compressed (this may be post mortem); head strongly wider than long (CI 1.19–1.33), mostly due to lateral extent of eyes; ocellar triangle extending to posterior head margin in full face view as a crown; occipital carinae distinct.

Mesosoma fairly slender (MSNI 1.56–1.80, WL 0.64–0.80); mesoscutum in dorsal view slightly wider than long; scutellum with two distinct faces: anterior face short and steeply sloping from mesoscutum, posterior face long and flat; scutellum in dorsal view oval and posteriorly rounded, but dorsoposterior margin carinate; dorsal face of propodeum about as long as posterior face; propodeal spines absent; petiole in dorsal view more or less rectangular, but carinae or denticles absent and all margins rounded, in lateral view petiole anteriorly tapering; anteroventral subpetiolar tooth absent; postpetiole globular, but dorsally somewhat compressed and flat, median impression absent; wings clear.

Sculpture smooth and shiny throughout; face with 2 longer erect setae close to ocelli and sparse short suberect pilosity; mesoscutum with scattered short erect or suberect pilosity; longer erect pilosity present on posterior part of scutellum; petiole with one pair of fine, erect setae; postpetiole with fine dorsoposterior setae. Head medium brown, mesosoma pale yellow, metasoma light brown.

##### Distribution and biology.

*Crematogaster rasoherinae* is the most widespread species of the Malagasy *Orthocrema*, and in fact it is one of the most abundant *Crematogaster* species in Madagascar and on a number of Indian Ocean islands (Figures 26 D–F). The species is distributed throughout all native forest habitats in Madagascar – rainforest, dry or spiny forest alike – and is also found in disturbed habitats and urban areas. It has been collected at elevations up to 1225m, but appears to be more common at lower elevations. In natural habitats this species is predominantly arboreal nesting, both in dead twigs as well as in live plant parts. However, ground nesting in rotten logs or branches does occur occasionally.

Biologically *Crematogaster rasoherinae* is interesting because of the presence of intermediate workers (i.e., individuals intermediate between workers and queens) (Figures 25 A–E) of unknown function in the colony. I have found intermediates in all four colonies that I collected of this species, with the highest number hereby being eight individuals in one nest. In all cases a normal, dealate queen also was present in the nest. None of the intermediates observed was winged and it seems likely that they are either entirely wingless or brachypterous. Scanning electron micrographs of the lateral mesosoma (Figures 25 C and D) show the presence of a rudimentary suture above the mesopleuron where in a normal queen the forewing attaches.

##### Discussion.

An intriguing characteristic of *Crematogaster rasoherinae* is the morphological variability of this species on the Comoros Islands compared to the remainder of its distribution range. In Madagascar, the Seychelles and Mayotte this species always possesses propodeal spines. On the Comoros Islands in contrast, propodeal spines can be present (as in Figure 24B), reduced or entirely absent (as in Figure 24E). More specifically, all specimens examined from the island of Grand Comore have no, or very reduced propodeal spines, whereas on Anjouan and Moheli propodeal spines are mostly reduced or absent and present only in fewer individuals. This spine-polymorphism was presumably the basis of the description of the here synonymized *Crematogaster voeltzkowi*. Anjouan is the type locality for this species name and the syntype specimens represent the morphological form lacking the propodeal spines. Analysis of DNA sequence data from both the nuclear markers (see Figure 1) and ancillary mitochondrial data however clearly shows a lack of genetic divergence between the ‘armed’ and ‘unarmed’ forms in *Crematogaster rasoherinae*. The cause and maintenance of this intraspecific polymorphism remains to be investigated.

The syntype specimens of *Crematogaster rasoherinae* have been lost or destroyed during the times of World War II. Confirmation for this has been obtained via e-mail communication with the Naturhistorisches Museum Hamburg (F. Wieland, 19.vii.2011). I designate a neotype in this study to unequivocally ascertain the identity of the species *Crematogaster rasoherinae*, hereby selecting a worker specimen from or close to the original type locality in Madagascar, Tamatave [Toamasina, town]. In a large and taxonomically difficult genus such as *Crematogaster* type material is indispensable to clarify species identities. Although no closely resembling species is currently known, it is likely that a morphologically similar species could be discovered in the future, either in Madagascar or on the African mainland.

### *Crematogaster madecassa-*group

**Worker diagnosis of the *Crematogaster madecassa*-group:** *Crematogaster madecassa*, *Crematogaster telolafy*, *Crematogaster razana*. Very small species (HW 0.48–0.60, WL 0.44–0.69). Masticatory margin of mandibles with 4 teeth; posterior margin of head in full face view usually laterally rounded, sometimes medially slightly depressed; occipital carinae well pronounced; antennal scape length variable; midline of eyes situated well above midline of head in full face view; eyes large (OI 0.22–0.28) and distinctly protruding.

Pronotum laterally subangular; mesonotum laterally with distinct, raised carinae that are confluent with lateral carinae bordering metanotal groove and propodeum; in lateral view outline of promesonotum moderately convex; mesonotum transversely concave, without a distinct posterior face and gradually sloping into metanotal groove; metanotal groove in dorsal view constricted by bordering lateral carinae, propodeal spines short to medium-sized (SPI 0.10–0.26), form variable; dorsal face of propodeum very short; petiole in dorsal view ovo-rectangular, with dorsolateral margins increasingly carinate posteriorly, ending in small posterolateral denticles; subpetiolar process variable: from small, but distinct and acute tooth to reduced angular dent; postpetiole globular, faintly impressed posteriorly, no trace of median impression; subpostpetiolar process present or absent.

Sculpture overall reduced; head shiny; mesosoma dorsally mostly shiny, carinulate laterally; meso- and metapleuron mostly shiny, with some reticulations; dorsal face of propodeum carinulate, posterior face shiny; dorsal face of petiole shiny; helcium dorsally carinulate; postpetiole dorsally feebly reticulate; lateral and ventral face of petiole and postpetiole reticulate; face with 4–8 erect, long flexuous setae, and abundant shorter, subdecumbent pubescence; promesonotum usually with 4–6 erect, long flexuous setae: 2 humeral setae, and 2 setae at anterior and usually also 2 setae at posterior end of mesonotal carinae; additional long erect setae, and scattered shorter erect setae may be present dorsally on promesonotum; petiole with a single stiff, erect seta on each posterolateral tubercle; postpetiole with a pair of erect dorsoposterior setae; abdominal tergites and sternites 4–7 with fairly abundant, erect long pilosity (> 20 setae) and sparse decumbent pubescence throughout. Color pale to medium yellow, or yellowish-brown.

**Queen diagnosis of the *Crematogaster madecassa*-group:** *Crematogaster madecassa*, *Crematogaster razana* (*Crematogaster telolafy* unknown). Very small (HW 0.80–1.10, WL 1.28–1.74). With worker characters, except as follows. Masticatory margin of mandibles with 5 teeth; antennal scapes not, or just reaching posterior margin of head; eyes large (OI 0.29–0.37) and protruding, situated slightly above midline of head in full face view; head wider than long (CI 1.11–1.21) and widest just posterior to eyes, posterior margin of head straight.

Mesosoma more compact (MSNI 1.55–1.82, WL 1.28–1.74); mesoscutum in dorsal view almost or as wide as long; dorsal face of propodeum absent, and posterior face very sharply and almost vertically sloping; propodeal spines present, much shorter than in workers (SPI 0.02–0.14), sometimes reduced to minute dents; petiole and postpetiole as in workers.

Sculpture smooth and shiny throughout, except metapleuron and anteriormost part of propodeum carinulate; erect pilosity very abundant on head, dorsal side of mesosoma and on metasoma, but finer and shorter than in workers; petiole with 1–3 pair(s) of long flexuous setae posterior to denticles; postpetiole with abundant erect pilosity. Color similar to respective workers, but often metasoma darker.

#### Crematogaster madecassa

Emery

http://species-id.net/wiki/Crematogaster_madecassa

Figures 27 28

Crematogaster sordidula var. *madecassa* Emery, 1895: 342. Worker and queen syntypes from MADAGASCAR: Diego-Suarez (Ch. Alluaud) [MSNG, examined]. Combination in *Crematogaster* (*Orthocrema*): Wheeler, W.M. 1922:1024. Subspecies of *sordidula*: Wheeler, W.M. 1922:1024. Raised to species: Emery, 1912: 668; Emery, 1922:131.

##### Type material examined

**(MSNG).** MADAGASCAR: *Antsiranana*: Diego-Suarez: [-12.26670, 49.28330] (Ch. Alluaud), CASENT0102053, CASENT0102054 and CASENT0101933. **Lectotype worker** by present designation: lower specimen of 2 workers on one pin, CASENT0102054 (image on AntWeb).

##### Other material examined

(CASC, PSWC, MSNG, MCZC). MADAGASCAR: *Antsiranana*: Sakalava Beach: -12.26278, 49.39750, 10m (R. Harin’Hala); 7 km N Joffreville: -12.33333, 49.25000, 360m (R. Harin’Hala); R.S. Ambre:-12.46889, 49.24217, 325m (B.L.Fisher et al.); P.N. Montagne d’Ambre: -12.50035, 49.17500, 885m; -12.53444, 49.17950, 925m (B.L.Fisher et al.); R.S. Manongarivo: -13.96167, 48.43333, 400m; -13.97667, 48.42333, 780m; -13.99833, 48.42833, 1175m (B.L.Fisher et al.); Ampasindava, Ambilanivy: -13.79861, 48.16167, 600m (B.L.Fisher et al.); Nosy Bé, R.N.I. Lokobé: -13.41944, 48.33117, 30m (B.L.Fisher et al.); F Andavakoera: -13.11833, 49.23000, 425m (B.L.Fisher et al.); F Antsahabe: -13.21167, 49.55667, 550m (B.L.Fisher et al.); F Binara: -13.25500, 49.61667, 375m; -13.26333, 49.60333, 650–800m (B.L.Fisher et al.); F Analabe: -13.08333, 49.90833, 30m (B.L.Fisher et al.); F Bekaraoka: -13.16667, 49.71000; 150m (B.L.Fisher et al.); F Ampondrabe: -12.97000, 49.70000, 175m (B.L.Fisher et al.); Montagne d’Akirindro: -15.28833, 49.54833, 600m (B.L.Fisher et al.); 6.9 km NE Ambanizana: -15.56667, 50.00000, 825m (B.L.Fisher et al.); Montagne d’Anjanaharibe: -15.18833, 49.61500, 470–1100m (B.L.Fisher et al.); P.N. Marojejy: -14.43333, 49.78333, 450m; -14.43817, 49.77400, 488m; -14.43500, 49.76000, 775m (B.L.Fisher et al.); P.N. Marojejy [Manantenina]: -14.43667, 49.77500, 450m (B.L.Fisher et al.); R.N.I. Marojejy: -14.43583, 49.76056, 610m (G. Alpert); F Ambanitaza: -14.67933, 50.18367, 240m (B.L.Fisher et al.); F Betaolana: -14.52996, 49.44039, 880m (B.L.Fisher et al.); P.N. Ankarana: -12.86361, 49.22583, 210m (B.L.Fisher); F Ambato: -13.46450, 48.55167, 150m (B.L.Fisher); F Anabohazo: -14.30889, 47.91433, 120m (B.L.Fisher et al.); 30km N Antalaha, Amboangy, -14.66480, 50.19070, 130m (G.Alpert); *Fianarantsoa*: P.N. Andringitra: -22.23333, 47.0000, 825m (B.L.Fisher et al.); F Vevembe: -22.79100, 47.18183, 600m (B.L.Fisher et al.); Rés. Marotandrano: -16.28322, 48.81443, 865m (B.L.Fisher et al.); R.S. Manombo: -23.01580, 47.71900, 30m (B.L.Fisher et al.); Mahabo [Rés. Forestière d’Agnalazaha]: -23.19383, 47.72300, 20m (B.L.Fisher et al.); *Mahajanga*: PN Ankarafantsika (Ampijora): -16.32083, 46.81067, 130m (B.L.Fisher et al.); *Toamasina*: P.N. Mananara-Nord: -16.45500, 49.78750, 225m (B.L.Fisher et al.); RS Ambatovaky: -16.81739, 49.29402, 360m; -16.77274, 49.26551, 450m; -16.81209, 49.29216, 460m; -16.77020, 49.26638, 470m; -16.76330, 49.26692, 520m (B.L.Fisher et al); F Ambatovy: -18.84950, 48.29470, 1010m; F.C. Sandranantitra: -18.04833, 49.09167, 450m (B.L.Fisher et al.); Rés. Betampona: -17.92400, 49.19967, 390m; -17.88667, 49.20250, 520m (B.L.Fisher et al.); F Kalalao [Ile St.Marie]: -16.92250, 49.88733, 100m (B.L.Fisher et al.); F Sahafina: -18.81445, 48.96205, 100m; Rés. Ambodiriana: -16.67233, 49.70117, 125m (B.L.Fisher et al.); Forêt d’Analava Mandrisy: -16.48567, 49.84700, 10m (B.L.Fisher et al.); S.F. Tampolo: -17.28250, 49.43000, 10m (B.L.Fisher et al.); *Toliara*: F Ivohibe: -24.56900, 47.20400, 200m (B.L.Fisher et al.); P.N. Andohahela: -24.75850, 46.85370, 275m (B.L.Fisher et al.); 10km NW Enakara, Rés. Andohahela: -24.56667, 46.81667, 430m; 11km NW Enakara, Rés. Andohahela: -24.56667, 46.83333, 800m (B.L.Fisher); 6km SSW Eminiminy, Rés. Andohahela: -24.75000, 46.78330, 500m (P.S.Ward); 9km SSW Eminiminy, Rés. Andohahela: -24.73330, 46.80000, 330m (P.S.Ward); 2.7km WNW 302º St.Luce: -24.77167, 47.17167, 20m (B.L.Fisher et al.); F Mandena: -24.95167, 47.00167, 20m (B.L.Fisher).

##### Worker measurements

**(n=21). Lectotype worker:** HW 0.54; HL 0.51; EL 0.13; SL 0.43; WL 0.54; SPL 0.13; PTH n.a.; PTL 0.17; PTW 0.16; PPL 0.10; PPW 0.17; LHT 0.40; CI 1.05; OI 0.26; SI 0.85; SPI 0.23; PTHI n.a.; PTWI 0.95; PPI 1.67; LBI 1.36.

##### Other material.

HW 0.48–0.60; HL 0.43–0.51; EL 0.10–0.14; SL 0.37–0.51; WL 0.44–0.63; SPL 0.08–0.14; PTH 0.09–0.14; PTL 0.14–0.22; PTW 0.12–0.22; PPL 0.09–0.14; PPW 0.13–0.21; LHT 0.33–0.52; CI 1.00–1.12; OI 0.22–0.27; SI 0.85–1.01; SPI 0.17–0.26; PTHI 0.55–0.72; PTWI 0.78–1.04; PPI 1.35–1.98; LBI 1.22–1.48.

##### Diagnosis.

Workers of *Crematogaster madecassa* can be distinguished from all other species treated here except *Crematogaster telolafy* by the presence of two distinct vertical carinae on the clypeus (Figure 16). From workers of *Crematogaster telolafy*, *Crematogaster madecassa* workers are distinguishable by their longer, more spiniform propodeal spines (Figure 18A) and longer antennal scapes. Queens of *Crematogaster madecassa* can be easily identified from queens of most species (*Crematogaster rasoherinae*, *Crematogaster volamena*, *Crematogaster mpanjono*) by the presence of propodeal spines. *Crematogaster madecassa* queens can be distinguished from *Crematogaster razana* queens by the absence of a median clypeal notch (present in *Crematogaster razana* queens). Note however that queens of *Crematogaster telolafy* are currently unknown and could be very similar morphologically to *Crematogaster madecassa* queens.

##### Worker description

(Figure 26A–C). Very small species (HW 0.48–0.60, WL 0.44–0.63), with characters of the *Crematogaster madecassa*-group, in addition to the following. Clypeus with two distinct median vertical carinae; antennal scapes well surpassing posterior margin of head.

Metanotal groove constricted to less than half as wide as pronotal width; propodeal spines medium-sized (SPI 0.17–0.26) and straight, usually thin and acute, in lateral view directed upwards, in dorsal view moderately diverging; subpostpetiolar process usually present, often as acute minute tooth.

Promesonotum usually with 6 erect, long flexuous setae: 2 humeral setae, and 2 setae each at anterior and posterior end of mesonotal carinae. Color pale to medium yellow.

##### Intermediate worker measurements

**(n=2).** HW 0.82–0.85, HL 0.73–0.78, EL 0.18–0.19, SL 0.55–0.59, WL 0.89–1.01, SPL 0.20, PTH 0.18–0.19, PTL 0.31–0.33, PTW 0.27–0.28, PPL 0.19, PPW 0.28–0.31, LHT 0.60, CI 1.09–1.12, OI 0.24, SI 0.75, SPI 0.20–0.22, PTHI 0.59, PTWI 0.86, PPI 1.46–1.66, LBI 1.48–1.68.

##### Intermediate worker description

**Figure 27.**
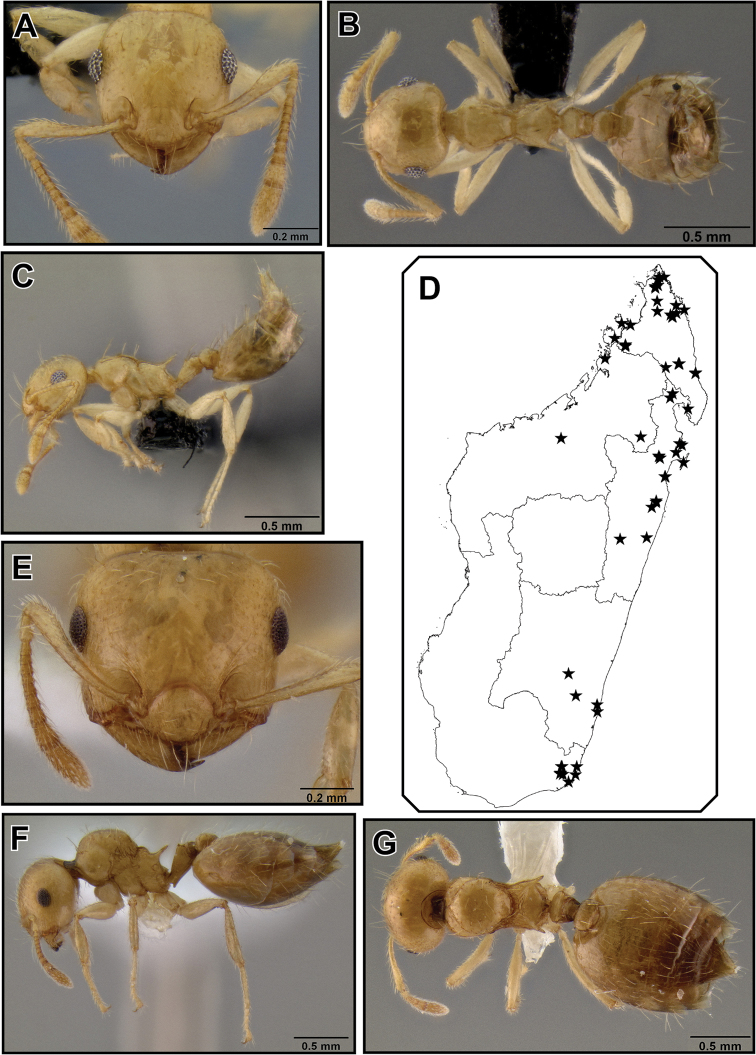
*Crematogaster madecassa*, worker, intermorph and distribution. **A–C** worker (CASENT0038498) **A** full face **B** dorsal **C** lateral **D** distribution **E–G** intermorph (CASENT0436247) **E** full face **F** lateral **G** dorsal.

(Figures 27E–G). Intermediate between workers and queens in size. Head, petiole and postpetiole characters similar to queens; ocelli present, but smaller than in queens; mesonotum is to various extent raised and fused dorsally over pronotum and has wing attachment sutures; otherwise mesosomal characters more similar to worker characters.

##### Queen measurements

**(n=11).** HW 0.87–1.03, HL 0.72–0.86, EL 0.24–0.32, SL 0.54–0.62, MSNW 0.66–0.92, MSNL 0.72–0.98, WL 1.28–1.53, SPL 0.06–0.21, PTH 0.19–0.26, PTL 0.38–0.49, PTW 0.30–0.39, PPL 0.21–0.28, PPW 0.34–0.42, LHT 0.65–0.81, CI 1.11–1.21, OI 0.29–0.37, SI 0.68–0.76, MSNI 1.55–1.87, SPI 0.04–0.14, PTHI 0.45–0.61, PTWI 0.68–0.91, PPI 1.42–1.69, LBI 1.87–2.08.

**Figure 28.**
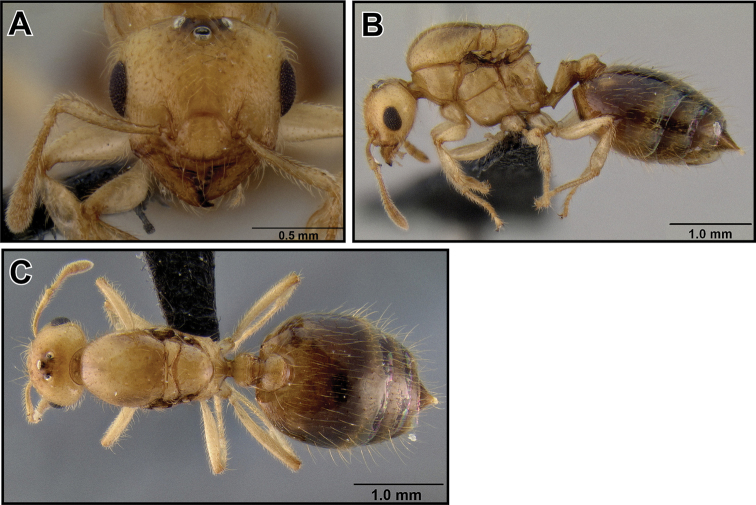
*Crematogaster madecassa*, queen. **A** full face, **B** lateral, **C** dorsal (CASENT0040391).

**Queen description** (Figure 28A–C). Very small (HW 0.87–1.03, WL 1.28–1.53), with characters of the *Crematogaster madecassa*-group, in addition to the following.

Mesosoma more compact (MSNI 1.55–1.87, WL 1.28–1.53), mesoscutum in dorsal view almost as wide as long; propodeal spines present, much shorter than in workers (SPI 0.04–0.14).

**Male** unknown.

##### Distribution and biology.

*Crematogaster madecassa* is fairly widespread throughout the rainforests and littoral forests of northern, eastern and southeastern Madagascar (Figure 27D) and is found up to medium elevations (highest record from 1175m). It occurs widely in sympatry with *Crematogaster rasoherinae*, and at a few localities also with *Crematogaster volamena*, *Crematogaster mpanjono* and *Crematogaster telolafy*. There is evidence that *Crematogaster madecassa* nests both on the ground and arboreally, as collections have been made from rotten logs, as well as from dead twigs above the ground. As in *Crematogaster rasoherinae* (see above), intermediate workers are found in *Crematogaster madecassa* (Figure 27E–F).

#### Crematogaster telolafy sp. n.

urn:lsid:zoobank.org:act:CC5E14B0-2EE5-42D7-847D-C7530A977114

http://species-id.net/wiki/Crematogaster_telolafy

Figure 29

##### Type locality.

MADAGASCAR: *Toliara*: P.N. Zombitse: -22.84333, 44.71000, 770m, tropical dry forest, sifted litter, 5–9.ii.2003, B.L.Fisher et al.

##### Type specimens.

**holotype** worker: pinned, CASENT0032779, BLF07510(19), sifted litter; original locality label: MADG’R: Prov. Toliara, P.N. Zombitse, 19.8 km 84°E Sakaraha 770m, 5–9.ii.2003, 22°50.6’S, 44°42.6’E, Fisher et al. BLF7510; deposited at CASC.

4 paratype workers: #1: pinned, CASENT0473872, BLF04605(29), spiny forest/thicket, ex dead tree stump; original locality label: MADG’R: Prov. Toliara, Kirindy, 15.5 km 64 ENE Marofandilia, 28.xi.–3.xii.2001, 100m 20°03'S, 44°40'E, Fisher et al. BLF4605; deposited at SAMC. #2: pinned, CASENT0473867, BLF04605(7), same habitat and label data as #1; deposited at MHNG. #3: pinned, CASENT0419808, BLF4434(7), tropical dry forest, ex rotten log; original locality label: MADG’R: Prov. Mahajanga, P.N. Tsingy de Bemaraha, 10.6 km 123°ESE Antsalova, 150m 18°43'S, 44°43'E, 16–20.xi.2001, Fisher et al. BLF4434; deposited at MCZC. #4: pinned, CASENT0193950, BLF04434(7), same habitat and label data as #3; deposited at UCDC.

##### Other material examined

(CASC). MADAGASCAR: *Fianarantsoa*: P.N. Isalo: -22.31333, 45.29167,500m; (B.L.Fisher et al.); F Analalava: -22.59167, 45.12833, 700m (B.L.Fisher et al.); *Mahajanga*: F Tsimembo-19.02139, 44.44067, 20m (B.L.Fisher et al.); P.N. Tsingy de Bemaraha: -19.14194, 44.82800, 50m; -19.13222, 44.81467, 100m; -18.70944, 44.71817, 150m (B.L.Fisher et al.); *Toliara*: R.S. Ambohijanahary: -18.26667, 45.40667, 1050m (B.L.Fisher et al.); Kirindy: -20.04500, 44.66222, 100m (B.L.Fisher et al.); P.N. Zombitse: -22.84333, 44.71000, 770m; -22.88650, 44.69217, 840m (B.L.Fisher et al.); P.N. Andohahela: -24.75850, 46.85370, 275m (B.L.Fisher et al.).

##### Worker measurements

**(n=16). Holotype:** HW 0.58; HL 0.55; EL 0.15; SL 0.44; WL 0.58; SPL 0.07; PTH 0.13; PTL 0.20; PTW 0.17; PPL 0.13; PPW 0.18; LHT 0.45; CI 1.06; OI 0.27; SI 0.80; SPI 0.13; PTHI 0.64; PTWI 0.82; PPI 1.34; LBI 1.29.

##### Other material.

HW 0.50–0.58; HL 0.47–0.56; EL 0.12–0.14; SL 0.39–0.46; WL 0.52–0.69; SPL 0.06–0.11; PTH 0.10–0.15; PTL 0.17–0.22; PTW 0.14–0.17; PPL 0.10–0.13; PPW 0.15–0.19; LHT 0.38–0.48; CI 1.02–1.09; OI 0.23–0.28; SI 0.78–0.87; SPI 0.10–0.19; PTHI 0.51–0.70; PTWI 0.67–0.89; PPI 1.22–1.60; LBI 1.24–1.67.

##### Diagnosis.

Workers of *Crematogaster telolafy* can be differentiated from all other Malagasy *Orthocrema* species except *Crematogaster madecassa* by the presence of two distinct vertical carinae on the clypeus (Figure 16). From *Crematogaster madecassa*, which shares this feature, *Crematogaster telolafy* workers are distinguished by the form (triangular) and the shorter length of their propodeal spines (Figures 18B and C), and by the shorter antennal scape. Queens of *Crematogaster telolafy* are unknown, but are expected to be morphologically similar to *Crematogaster madecassa* queens.

##### Worker description

**Figure 29.**
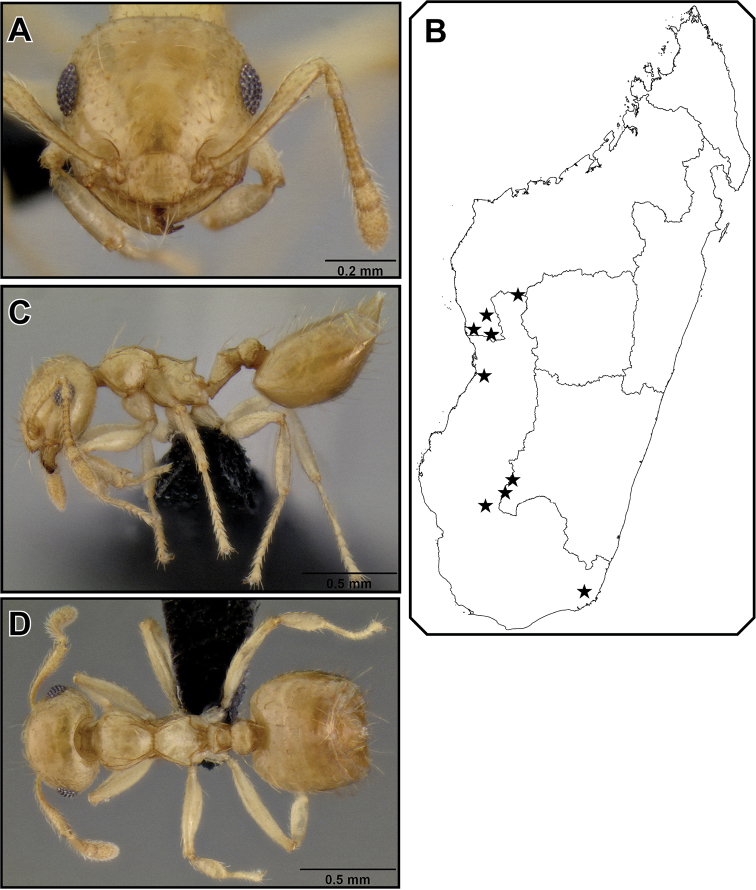
*Crematogaster telolafy*, worker and distribution. **A** full face **B** distribution **C** lateral **D** dorsal (CASENT0032779).

(Figure 29A, C–D). Very small species (HW 0.50–0.58, WL 0.52–0.69), with characters of the *Crematogaster madecassa*-group, in addition to the following. Clypeus with two distinct median vertical carinae; antennal scapes reaching, or barely surpassing posterior margin of head.

Metanotal groove constricted to less than half as wide as pronotal width; propodeal spines short to medium-sized (SPI 0.10–0.19), usually in form of acute triangular points, sometimes more elongate and spiniform, distinctly directed upwards in lateral view, in dorsal view parallel or moderately diverging; subpostpetiolar process usually present, often as acute minute tooth.

Promesonotum usually with 6 erect, long flexuous setae: 2 humeral setae, and 2 setae each at anterior and posterior end of mesonotal carinae. Color pale to medium yellow.

##### Queen, male and intermediate

(if existing)unknown.

##### Distribution and biology.

No colony collections of *Crematogaster telolafy* have been made that could give clues on the biology of this species. It has been collected by methods of litter sifting, malaise, pitfall and pan traps, as well as beating low vegetation. *Crematogaster telolafy* is distributed mainly in the dry and spiny forests of southern and western Madagascar at low elevations (Figure 29B), with some notable exceptions of records from remnant patches of western rainforest, e.g. Rés. Ambohijanahary, or gallery forest such as exists in the Isalo region. This species is allopatric with the closely related *Crematogaster madecassa* (see above), except for a narrow sympatry or parapatry in the Andohahela region, where one collection of *Crematogaster telolafy* has been made in low elevation rainforest. Otherwise *Crematogaster telolafy* occurs in sympatry only with *Crematogaster rasoherinae* among the species treated here.

##### Etymology.

This species is named for the triangular form of its propodeal spines, as “telolafy” is the Malagasy word for triangle. This name should be treated as a noun in apposition.

#### Crematogaster razana sp. n.

urn:lsid:zoobank.org:act:F8F5FEA8-5783-46B8-A9A8-8DFF42EBB778

http://species-id.net/wiki/Crematogaster_razana

Figure 30

##### Type locality.

MADAGASCAR: *Toliara*: R.S. Kalambatritra: -23.4185, 46.4583, 1365m, grassland, under stone; 8.ii.2009; B.L.Fisher et al..

##### Type specimen.

**holotype** worker:pinned, CASENT0149655, BLF21485; original locality label: MADG’R: *Toliara*: R.S. Kalambatritra: 23.4185°S, 46.4583°E, 1365m; grassland, 8.ii.2009; B.L.Fisher et al. BLF#; deposited at CASC.

##### Other material examined

(CASC). MADAGASCAR: *Toliara*: R.S. Kalambatritra: -23.45373, 46.45773, 1345m; -23.4185, 46.4583, 1365m (B.L.Fisher et al.); P.N. Andohahela: -24.9300, 46.6455, 300m (B.L.Fisher et al.).

##### Worker measurements

**(n=3). Holotype:** HW 0.54; HL 0.50; EL 0.13; SL 0.37; WL 0.53; SPL 0.07; PTH 0.12; PTL 0.18; PTW 0.15; PPL 0.12; PPW 0.16; LHT 0.38; CI 1.08; OI 0.26; SI 0.74; SPI 0.14; PTHI 0.68; PTWI 0.86; PPI 1.31; LBI 1.40.

##### Other material.

HW 0.49–0.56; HL 0.45–0.52; EL 0.12–0.14; SL 0.35–0.40; WL 0.45–0.54; SPL 0.05–0.07; PTH 0.10–0.12; PTL 0.15–0.19; PTW 0.14–0.16; PPL 0.08–0.11; PPW 0.14–0.17; LHT 0.34–0.40; CI 1.07; OI 0.26–0.27; SI 0.76–0.77; SPI 0.11–0.13; PTHI 0.64–0.67; PTWI 0.86–0.92; PPI 1.46–1.70; LBI 1.32–1.37.

##### Diagnosis.

A combination of protruding eyes and raised sharp lateral carinae on the propodeum (Figure 13) separates workers of *Crematogaster razana* from workers of *Crematogaster rasoherinae* and the *Crematogaster volamena*-group. From other species within the *Crematogaster madecassa*-group it can be identified by the lack of median vertical carinae on the clypeus (Figure 17) and the absence of long setae on the posterior end of the lateral mesonotal carinae. *Crematogaster razana* queens are diagnosed by a combination of the presence of very short propodeal spines, large protruding eyes (OI 0.31), and the presence of a median clypeal notch (Figure 20).

##### Worker description

**Figure 30.**
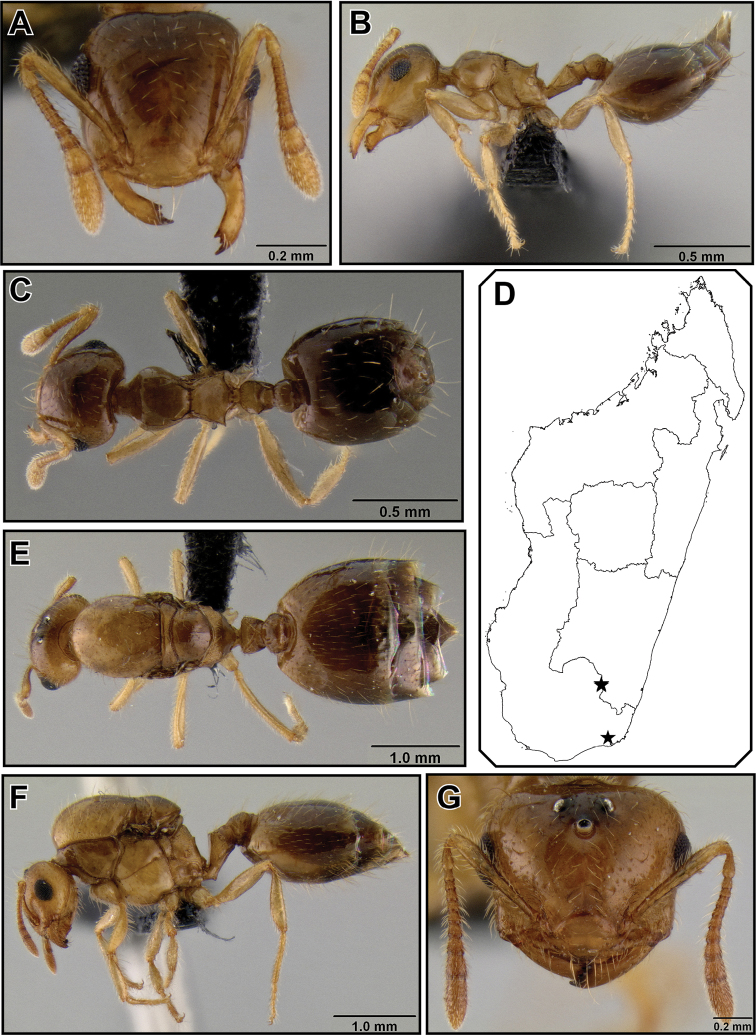
*Crematogaster razana*, worker, queen and distribution. **A–C** worker (CASENT0149655) **A** full face **B** lateral **C** dorsal **D** distribution **E–G** queen (CASENT0148782) **E** dorsal **F** lateral **G** full face.

(Figures 30A–C). Very small species (HW 0.49–0.56, WL 0.45–0.54), with characters of the *Crematogaster madecassa*-group, in addition to the following. Clypeus lacking median vertical carinae; antennal scapes just reaching posterior margin of head.

Metanotal groove constricted to about half the width of pronotum; propodeal spines short (SPI 0.11–0.14), in form of acute triangular points, distinctly directed upwards in lateral view, in dorsal view moderately diverging; subpostpetiolar process absent.

Face with no more than 4 erect, long flexuous setae; promesonotum with 4 erect, long flexuous setae: 2 humeral setae, and 2 setae at anterior end of mesonotal carinae (posterior setae absent). Color yellow to yellowish-brown.

##### Queen measurements

**(n=1).** HW 1.10, HL 0.91, EL 0.28, SL 0.65, MSNW 0.94, MSNL 0.98, WL 1.74, SPL 0.04, PTH 0.30, PTL 0.45, PTW 0.41, PPL 0.28, PPW 0.46, LHT 0.83, CI 1.21, OI 0.31, SI 0.71, MSNI 1.77, SPI 0.02, PTHI 0.66, PTWI 0.92, PPI 1.65, LBI 2.09.

##### Queen description

(Figures 30E–G). Very small (HW 1.10, WL 1.74), with characters of the *Crematogaster madecassa*-group, in addition to the following. Clypeus with a median notch; antennal scapes just reaching posterior margin of head.

Mesosoma more compact (MSNI 1.77, WL 1.74); mesoscutum in dorsal view about as wide as long; propodeal spines reduced to minute dents (SPI 0.02).

Petiole with one pair of long flexuous setae posterior to denticles.

##### Male and intermediate

(if existing) unknown.

##### Distribution and biology.

*Crematogaster razana* has only been collected at two localities in southern Madagascar (Figure 30B). One of these sites is a dry forest (western slopes of P.N. Andohahela), whereas the other collections were made in a montane grassland habitat (R.S. Kalambatritra). Since the few collections of this rare species have been made mostly on the ground (under stone, pitfall trap or on low vegetation), I assume that *Crematogaster razana* is ground-nesting. This species is sympatric with *Crematogaster rasoherinae* and parapatric or narrowly sympatric with both *Crematogaster madecassa* and *Crematogaster telolafy* in the Andohahela massif.

##### Etymology.

TheMalagasy word “razana” means ancestor and alludes to the isolated distribution of this species and its phylogenetic position within the *Crematogaster madecassa*-group. This name should be treated as a noun in apposition.

### *Crematogaster volamena*-group

**Worker diagnosis of the *Crematogaster volamena*-group**: *Crematogaster volamena*, *Crematogaster mpanjono*.

Very small to medium sized species (HW 0.51–0.98, WL 0.56–0.92). Masticatory margin of mandibles with 4 teeth; clypeus with or without several irregular vertical carinae; posterior margin of head in full face view laterally subangular, often medially slightly depressed; occipital carinae indistinct; antennal scapes just barely (small workers) or not reaching (larger workers) posterior margin of head; midline of eyes situated well above midline of head in full face view; eyes small (0.18–0.22) and fairly flush with head.

Pronotum laterally subangular; in lateral view, anterior part of mesonotum often angular or denticulate, posteriorly at least weakly carinate until meeting metanotal groove; in lateral view outline of promesonotum fairly flat; dorsal face of mesonotum flat, posterior face distinct or indistinct; metanotal groove very constricted by bordering lateral carinae, a third as wide as pronotal width; propodeal spines short (SPI 0.06–0.12), upwards directed sharp points; length of dorsal face of propodeum about a third of posterior face; petiole in dorsal view ovo-rectangular, dorsolateral margins angulate, ending in small posterolateral denticles; subpetiolar process variable, from well pronounced acute tooth to reduced angular dent; postpetiole short and broad, appearing bilobed, with diffuse, broad median impression; subpostpetiolar process absent.

Sculpture overall reduced; head shiny to aciculate; mesosoma dorsally mostly shiny, meso- and metapleuron mostly shiny, rugulose in some parts; dorsal and posterior face of propodeum shiny with some carinulae; dorsal face of petiole shiny to carinulate; helcium dorsally reticulate; postpetiole dorsally shiny; lateral and ventral face of petiole and postpetiole feebly reticulate; face with very abundant silken erect to suberect pilosity of variable length, usually hereof 6–12 longer setae; promesonotum with at least 6 erect, long flexuous setae: 2 humeral setae, 2 setae at anterior end of mesonotum and 2 setae on mesonotal tubercles or denticles; additional long erect setae, and scattered shorter erect setae may be present dorsally on promesonotum; longer, erect pilosity present or absent from propodeum; petiole with a single flexuous setae on each posterolateral tubercle; postpetiole with a pair of long flexuous dorsoposterior setae, and several shorter setae dorsally and laterally; abdominal tergites and sternites 4–7 with dense erect pilosity (> 50 setae) of medium length, interspersed with a subdecumbent shorter pubescence. Color, pale or golden yellow, or medium brown.

**Queen diagnosis of the *Crematogaster volamena*-group**: *Crematogaster volamena*, *Crematogaster mpanjono*.

Large (HW 1.48–1.72, WL 2.61–2.70). With worker characters, except as follows. Masticatory margin of mandibles with 5 teeth. Antennal scapes not surpassing posterior margin of head, reaching to about level of median or lateral ocelli; occipital carinae well pronounced or indistinct; eyes medium-sized to large (OI 0.23–0.27), situated at midline of head in full face view; head wider than long or slightly longer than wide, posterior margin of head straight.

Mesosoma compact to slender (MSNI 1.64–1.77, WL 2.61–2.70); propodeal spines absent; petiole oval or subquadrate and lacking denticles; postpetiole broad, but lacking median impression; broad subpetiolar process present, but lacking distinct tooth.

Head sculpture aciculate or carinulate-aciculate; sculpture on mesosoma and metasoma aciculate, except dorsal face of propodeum transversely carinulate and metapleuron carinulate. Erect pilosity somewhat less abundant than in workers, but denser on mesoscutum and scutellum; petiolar and postpetiolar pilosity as in workers. Color brown with yellow markings on meso-, metasoma and legs, or reddish brown.

#### Crematogaster volamena sp. n.

urn:lsid:zoobank.org:act:916726B2-C7EB-49E0-BB6A-012F0C775E13

http://species-id.net/wiki/Crematogaster_volamena

Figures 31 32

##### Type locality.

MADAGASCAR: *Toliara*: Forêt Ivohibe: -24.56900, 47.20400, 200m, rainforest, malaise trap, 2.-4.xii.2006, B.L.Fisher et al.

##### Type specimens.

**holotype** worker: pinned, CASENT0125748, BLF15448, malaise trap; original locality label: MADG’R: Toliara, Forêt Ivohibe, 200m, 24°34.14S, 47°12.24E, 2–4.xii.06, rainforest, Fisher et al. BLF15448; deposited at CASC.

4 paratype workers: #1: pinned, CASENT0128455, BLF15450, sifted litter (leaf mold, rotten wood); same locality data as holotype; deposited at SAMC. #2: pinned, CASENT0488904, BLF08006(23), beating low vegetation, rainforest; original locality label: MADG’R: Prov. Toamasina, Mont. Anjanaharibe, 18.0 km, 21° NNE Ambinanitelo 470m 15°11.3'S, 49°36.9'E, 8–12.iii.2003 Fisher et al. BLF8006; deposited at MHNG. #3: pinned, CASENT0071334, BLF12557, malaise trap; original locality label: MADG’R: Prov. Toamasina, P.N. Mananara-Nord, 16°27.3'S, 49°47.25'E, 225m, 14.xi.2005, malaise, rainforest, Fisher et al. BLF12557; deposited at MCZC. #4: pinned, CASENT0488765, BLF8251(4), beating low vegetation; original locality label: MADG’R: Prov. Toamasina, Mont. Akirindro 7.6 km 341° NNW Ambinanitelo 15°17.3'S, 49°32.9'E 600m, 17–21.iii.2003, Fisher et al. BLF8251; deposited at UCDC.

##### Other material examined

(CASC, MCZC). MADAGASCAR: *Antsiranana*: P.N. Montagne d’Ambre: -12.52830, 49.17250, 1046m (D.Lees et al.); 6.9 km NE Ambanizana: -15.56667, 50.00000, 825m (B.L.Fisher et al.); 6.3 km S Ambanizana: -15.68131, 49.9580, 25m (B.L.Fisher et al.); 5.3 km SSE Ambanizana, 425m, -15.66667, 49.96667 (B.L.Fisher et al.); Montagne d’Anjanaharibe: -15.18833, 49.61500, 470m (B.L.Fisher et al.); P.N. Marojejy: -14.43333, 49.78333, 450m; -14.43817, 49.77400, 488m; -14.43500, 49.76000, 775m (B.L.Fisher et al.); R.N.I. Marojejy: -14.43583, 49.76056, 610m (G. Alpert); *Fianarantsoa*: F Ampitavananima: -23.12972, 47.71700, ca. 35m (B.L.Fisher et al.); *Toamasina*: R.S. Ambatovaky: -16.77468, 49.26551, 355m; -16.81745, 49.29250, 400m; -16.77550, 49.26427, 430m; -16.77274, 49.26551, 450m; -16.76912, 49.26704, 475m; -16.76330, 49.26692, 520m (B.L.Fisher et al); P.N. Mananara-Nord: -16.45500, 49.78750, 225m (B.L.Fisher et al.); F.C. Sandranantitra: -18.04833, 49.09167, 450m (B.L.Fisher et al.); Rés. Betampona: -17.91801, 49.20074, 500m (B.L.Fisher et al.); *Toliara*: F Ivohibe: -24.56900, 47.20400, 200m (B.L.Fisher et al.).

##### Worker measurements

**(n=20). Holotype:** HW 0.62; HL 0.59; EL 0.11; SL 0.50; WL 0.66; SPL 0.06; PTH 0.13; PTL 0.20; PTW 0.20; PPL 0.10; PPW 0.21; LHT 0.49; CI 1.04; OI 0.19; SI 0.85; SPI 0.09; PTHI 0.65; PTWI 0.99; PPI 2.07; LBI 1.35.

##### Other material.

HW 0.66–0.98; HL 0.62–0.95; EL 0.12–0.18; SL 0.50–0.66; WL 0.68–0.92; SPL 0.05–0.09; PTH 0.13–0.19; PTL 0.21–0.30; PTW 0.21–0.30; PPL 0.11; PPW 0.20–0.32; LHT 0.42–0.72; CI 1.03–1.13; OI 0.18–0.22; SI 0.73–0.84; SPI 0.06–0.12; PTHI 0.50–0.72; PTWI 0.82–1.08; PPI 1.43–2.13; LBI 1.25–1.71.

##### Diagnosis.

Workers of *Crematogaster volamena* are diagnosed most easily by their indistinct occipital carinae (Figure 14), the non-protruding and small eyes and the absence of raised, sharp lateral carinae on the propodeum (Figure 15). All these characteristics are shared with the much rarer, but closely resembling *Crematogaster mpanjono*, from which it cannot be distinguished reliably based on the worker caste. *Crematogaster volamena* has slightly longer propodeal spines than *Crematogaster mpanjono*, and the propodeum is lacking longer erect pilosity. The two species are not known to co-occur, and therefore their distributions (compare Figures 32F and 33D) can help in distinguishing between them. Queens of *Crematogaster volamena* are readily separated from *Crematogaster mpanjono* queens by virtue of their well pronounced occipital carinae (Figure 22) and a scuto-scutellar suture that is broadly meeting the mesoscutum (Figure 21, compare with Figure 23). From the remaining Malagasy *Orthocrema* queens, *Crematogaster volamena* queens are differentiated by the absence of propodeal spines and their large size (HW 1.72, WL 2.61).

**Figure 31.**
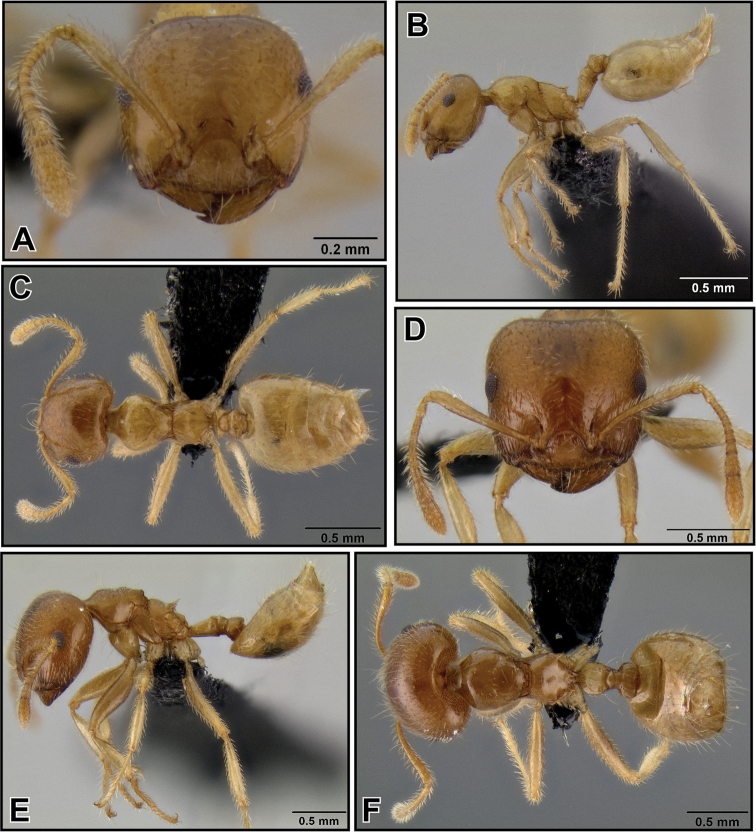
*Crematogaster volamena*, workers. **A–C** smaller worker (CASENT0125748) **A** full face **B** lateral **C** dorsal **D–F** larger worker (CASENT0122851) **D** full face **E** lateral **F** dorsal.

##### Worker description

(Figures 31A–F). Very small to medium sized species (HW 0.62–0.98, WL 0.66–0.92), with characters of the *Crematogaster volamena*-group, in addition to the following. Clypeus with several (up to 6), irregular vertical carinae.

Mesonotum transversely concave between lateral carinae.

Head sculpture aciculate; longer, erect pilosity absent from propodeum. Color golden yellow or (more rarely) medium brown.

##### Queen measurements

**(n=1).** HW 1.72, HL 1.60, EL 0.37, SL 0.90, MSNW 1.28, MSNL 1.59, WL 2.61, SPL 0.00, PTH 0.39, PTL 0.67, PTW 0.61, PPL 0.43, PPW 0.65, LHT 1.21, CI 1.08, OI 0.23, SI 0.56 , MSNI 1.64, SPI 0.15, PTHI 0.59, PTWI 0.91, PPI 1.50, LBI 2.16.

##### Queen description

**Figure 32.**
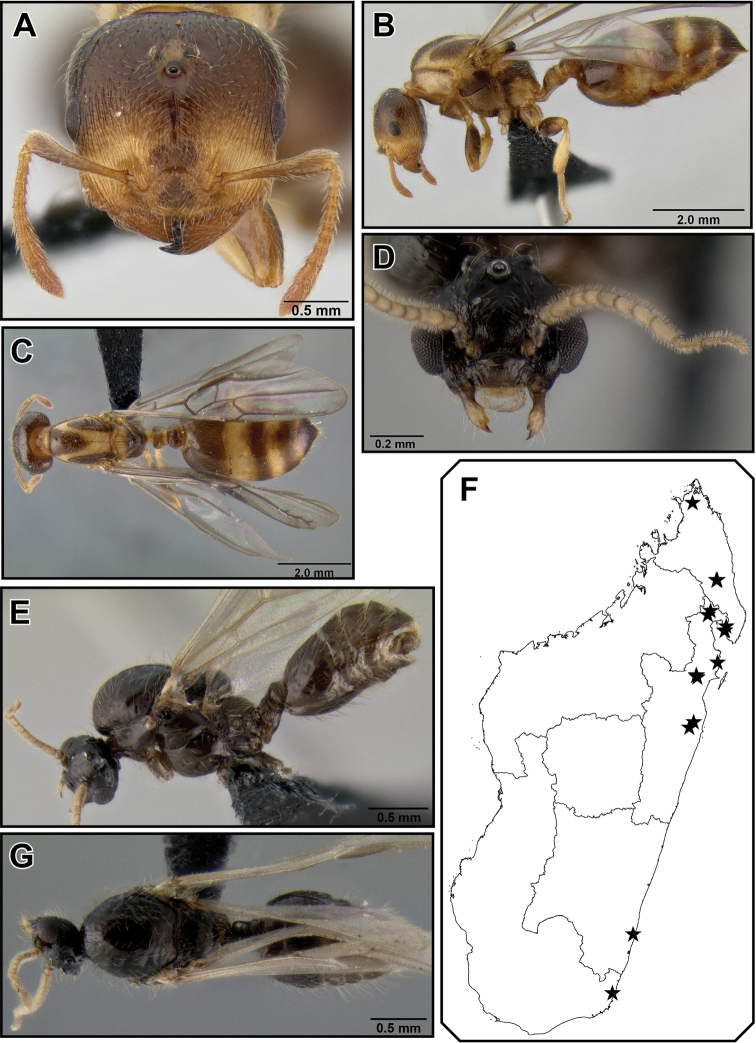
*Crematogaster volamena*, queen, male and distribution. **A–C** queen (CASENT0161415) **A** full face **B** lateral **C** dorsal **D–E,G** male (CASENT0162852) **D** full face **E** lateral **G** dorsal **F** distribution.

(Figures 32A–C). Large (HW 1.72, WL 2.61), with characters of the *Crematogaster volamena*-group, in addition to the following. Antennal scapes reaching to about level of median ocelli; occipital carinae well pronounced; eyes medium-sized (OI 0.23); head wider than long (CI 1.08).

Mesosoma compact (MSNI 1.64, WL 2.61); mesoscutum in dorsal view oval; scuto-scutellar suture broadly meeting mesoscutum; dorsal face of propodeum short but distinct, posterior face sloping abruptly; petiole in dorsal view oval; wings smoky.

Head sculpture carinulate-aciculate. Color brown with yellow markings on meso-, metasoma and legs.

##### Male measurements

**(n=1).** HW 0.68, HL 0.52, EL 0.26, SL 0.11, MSNW 0.73, MSNL 0.75, WL 1.44, SPL 0.00, PTH 0.19, PTL 0.31, PTW 0.22, PPL 0.15, PPW 0.26, LHT n.a., CI 1.30, OI 0.50, SI 0.21, MSNI 1.93, SPI 0.62, PTHI 0.62, PTWI 0.71, PPI 1.71, LBI n.a.

##### Male description 

(Figures 32 D,E–G). Small (HW 0.68, WL 1.44). Masticatory margin of mandibles with 2 teeth; eyes large (OI 0.50) and protruding, situated slightly below midline of head, and not approaching clypeal margin; antennae 11–12-segmented (separation between 3^rd^ and 4^th^ funicular segment is incomplete in the examined specimen), scapes very short (SI 0.21), 2^nd^ funicular segment globular, last 2 or 3 funicular segments compressed (this may be post mortem); head strongly wider than long (CI 1.30), mostly due to lateral extent of eyes; ocellar triangle extending to posterior head margin in full face view like a crown; occipital carinae distinct.

Mesosoma compact (MSNI 1.93, WL 1.44); mesoscutum in dorsal view as wide as long; scutellum with only one long and flat dorsal face, in dorsal view oval-shaped and posteriorly rounded, dorsoposterior margin not carinate; dorsal face of propodeum about as long as posterior face; propodeal spines absent; petiole in dorsal view oval, carinae or denticles absent and all margins rounded, in lateral view petiole anteriorly tapering; anteroventral subpetiolar tooth absent; postpetiole fairly globular, median impression absent; wings clear.

Head sculpture rugulose, mesoscutum aciculate, scutellum longitudinally carinulate, petiole and postpetiole rugulose-shiny; face with fairly abundant longer erect pilosity; mesoscutum with dense long erect pilosity; posterior part of scutellum with sparse long pilosity; petiole and postpetiole without distinct dorsoposterior setae, but abundant erect pilosity laterally. Color dark brown.

##### Distribution and biology.

*Crematogaster volamena* is currently known from about 10 localities along the eastern rainforest belt in Madagascar (Figure 32F). Here the species occurs in low- to mid-elevation rainforest or littoral forest. *Crematogaster volamena* appears to be a generalist in terms of nesting preferences, as it has been collected both nesting arboreally in dead twigs and on the ground in rotten logs. Scant natural history information exists, but noteworthy is a size variation in workers of this species that is reminiscent of a major-minor distinction in other ant genera. The larger workers (Figures 31D–F) have a distinctly enlarged head and more powerful mandibles compared to regular workers (Figures 31A–C), and they are more rarely seen throughout collections. These individuals do not share the attributes of the intermediates described here for *Crematogaster rasoherinae* and *Crematogaster madecassa*, such as presence of ocelli, or a queen-like modified mesosoma.

##### Etymology.

This species is named for the golden yellow coloration that most of its workers possess, as “volamena” means “gold” in Malagasy. This name should be treated as a noun in apposition.

#### Crematogaster mpanjono sp. n.

urn:lsid:zoobank.org:act:7A4E5257-126C-460D-B9A4-7C52CB2D62F1

http://species-id.net/wiki/Crematogaster_mpanjono

Figure 33

##### Type locality.

MADAGASCAR: *Antsiranana*: R.S. Manongarivo: -13.96167, 48.43333, 400m, rainforest, beating low vegetation, 18.xi.1998, B.L.Fisher.

##### Type specimen.

**holotype** worker: pinned, CASENT0193889, BLF01998(12)-1; original locality label: MADG’R: Prov. Antsiranana: R.S. Manongarivo 10.8km 229°SW Antanambao 400m 13°57.7'S, 48°26.0'E, 18.xi.1998, B.L.Fisher#1998(12)-1; deposited at CASC.

##### Other material examined

(CASC)

##### .

MADAGASCAR: *Antsiranana*: R.S. Manongarivo: -13.96167, 48.43333, 400m (B.L.Fisher); Nosy Bé, R.N.I. Lokobé: -13.41944, 48.33117, 30m (B.L.Fisher et al.); Nosy Bé, Antsirambazaha: -13.41345, 48.31130, 143m (Lees & Ranaivosolo); *Toamasina*: F Ambohidena [Ile St.Marie]: -16.82433, 49.96417, 20m (B.L.Fisher et al.).

##### Worker measurements

**(n=4). Holotype:** HW 0.61; HL 0.56; EL 0.11; SL 0.48; WL 0.65; SPL 0.06; PTH 0.15; PTL 0.22; PTW 0.19; PPL 0.13; PPW 0.21; LHT 0.46; CI 1.09; OI 0.20; SI 0.85; SPI 0.09; PTHI 0.69; PTWI 0.88; PPI 1.62; LBI 1.40.

##### Other material.

HW 0.51–0.78; HL 0.51–0.78; EL 0.10–0.15; SL 0.43–0.54; WL 0.56–0.86; SPL 0.04–0.05; PTH 0.11–0.16; PTL 0.17–0.27; PTW 0.16–0.23; PPL 0.09–0.15; PPW 0.19–0.22; LHT 0.39–0.56; CI 1.02–1.04; OI 0.19–0.20; SI 0.72–0.85; SPI 0.06–0.07; PTHI 0.60–0.63; PTWI 0.87–1.03; PPI 1.46–2.03; LBI 1.42–1.52.

##### Diagnosis.

**Figure 33.**
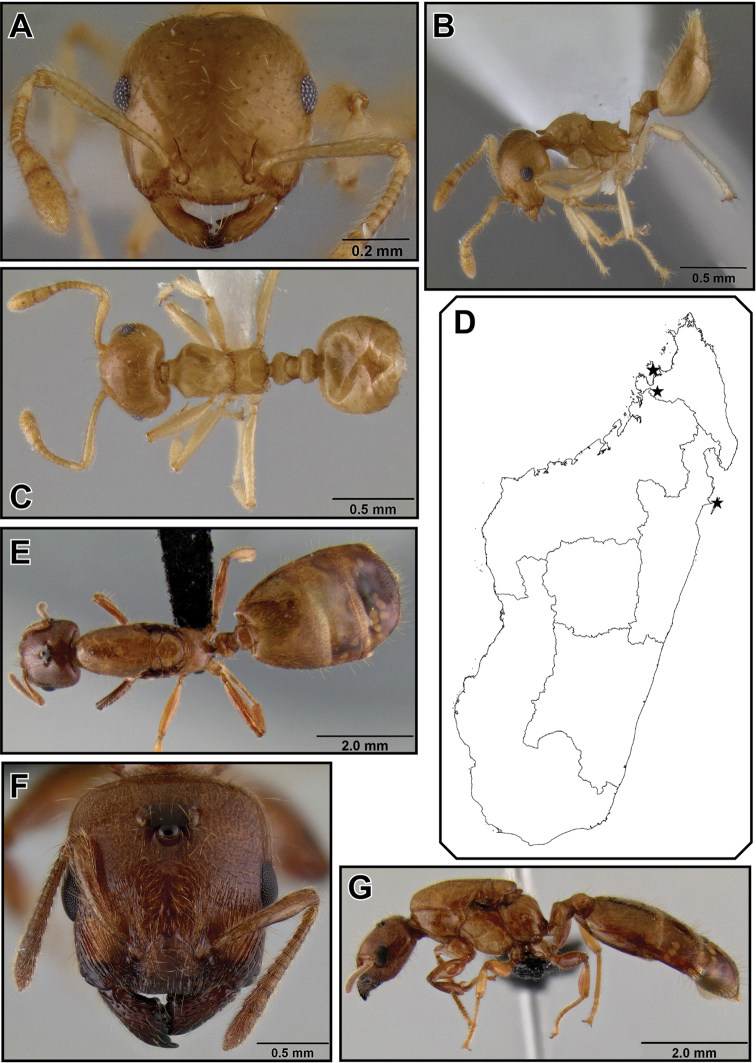
*Crematogaster mpanjono*, worker and queen. **A–C** worker (CASENT0193889) **A** full face **B** lateral **C** dorsal **D** distribution **E–G** queen (CASENT0067033) **E** dorsal **F** full face **G** lateral.

Workers of *Crematogaster mpanjono* are diagnosed from all Malagasy *Orthocrema*, except the closely related *Crematogaster volamena*, by a combination of the following: indistinct occipital carinae (Figure 14), non-protruding and small eyes and the absence of raised, sharp lateral carinae on the propodeum (Figure 15). All these characteristics are shared with the much more common *Crematogaster volamena*, from which it cannot be distinguished reliably based on the worker caste. *Crematogaster mpanjono* has slightly shorter propodeal spines than *Crematogaster volamena*, and often a long erect pilosity is present on the propodeum. The distributions of these two species (compare Figures 32F and 33D) aid in distinguishing between them, as they are not known to co-occur. Queens of *Crematogaster mpanjono* are easily separated from *Crematogaster volamena* queens by virtue of the scuto-scutellar suture that is acutely meeting the mesoscutum (Figure 23, compare with Figure 21) and the indistinct occipital carinae. From the remaining Malagasy *Orthocrema* queens, *Crematogaster mpanjono* queens are diagnosable by the absence of propodeal spines and by their larger size (HW 1.48, WL 2.70).

##### Worker description

(Figures 33A–C).Very small to small species (HW 0.51–0.78, WL 0.56–0.86), with characters of the *Crematogaster volamena*-group, in addition to the following. Clypeus with or without several short vertical carinae.

Mesonotum usually denticulate, then weakly carinate until meeting metanotal groove; posterior face of mesonotum indistinct, gradually sloping into metanotal groove.

Head sculpture shiny to aciculate; propodeum often with longer erect pilosity. Color yellow to pale yellow.

##### Queen measurements

**(n=1).** HW 1.48, HL 1.52, EL 0.42, SL 0.81, MSNW 1.04, MSNL 1.53, WL 2.70, SPL 0.00, PTH 0.39, PTL 0.51, PTW 0.51, PPL 0.39, PPW 0.53, LHT 1.07, CI 0.96, OI 0.27, SI 0.53, MSNI 1.77, SPI 0.00, PTHI 0.78, PTWI 1.01, PPI 1.35, LBI 2.52.

##### Queen description

(Figures 33E–G). Medium-sized (HW 1.48, WL 2.70), with characters of the *Crematogaster volamena*-group, in addition to the following. Antennal scapes reaching to about level of lateral ocelli; occipital carinae indistinct; eyes larger (OI 0.27); head slightly longer than wider (CI 0.96).

Mesosoma slender (MSNI 1.77, WL 2.70); mesoscutum in dorsal view elongate; scuto-scutellar suture acutely meeting mesoscutum; dorsal face of propodeum about as long as posterior face, the latter sloping abruptly; petiole subquadrate.

Head sculpture mostly aciculate, carinulate below eyes. Color reddish brown.

**Male** unknown.

##### Distribution and biology.

Only one nest collection of this species (a queen and a nanitic worker) from an arboreal root pocket exists. Therefore next to nothing is known about the biology of *Crematogaster mpanjono*. The known distribution records of this rare ant show a macrohabitat preference for lowland rainforests of the north-western Sambirano region (Nosy Bé, R.S. Manongarivo) or eastern littoral rainforest (F Ambohidena) (Figure 33D). These disjunct records from north-western Madagascar and the east coast island Ile St.Marie are peculiar and could point to incomplete distribution records for this species. In any case, although the Ambohidena population has not been sampled for nuclear genetic data, the conspecificity of these disjunct populations is supported by the COI barcoding data. *Crematogaster mpanjono* occurs in sympatry with the widespread *Crematogaster rasoherinae* and *Crematogaster madecassa*.

##### Etymology.

The Malagasy word “mpanjono” means “fisher “or “fisherman”. The name for this rare ant species is dedicated to B. L. Fisher and his ant diversity and conservation efforts in Madagascar. This name should be treated as a noun in apposition.

## Discussion

The species diversity of Malagasy *Crematogaster* (*Orthocrema*) has been tripled in the context of this revisionary work, adding four new species to two already described and here well supported species.Another result that was strongly supported by the molecular part of this study is the presence of three phylogenetically distinct lineages of Malagasy *Orthocrema* (Figure 1). The relationships between the *Crematogaster madecassa*- and *Crematogaster volamena*-group remain weakly supported, similarly to results of a larger analysis (Blaimer, in prep.). The molecular data, as well as distinctive morphological differences indicate that these two species-groups do not constitute a monophyletic group. One can therefore assume they reached Madagascar through independent colonization events.

In analogy to previous taxonomic studies on the genus in Madagascar (Blaimer 2010; in prep.), a widespread species, *Crematogaster rasoherinae*, has been found to have synonymic names. Widespread species in *Crematogaster* are prone to this “over-naming” as they can show strong gradual geographic variation. Discrete polymorphisms, as in the case of the entirely unarmed form of *Crematogaster rasoherinae* on the Comoros Islands, are presumably much rarer, but another case of strong polymorphism has just been described for queens of *Crematogaster ranavalonae* Forel in Madagascar (Blaimer 2012). In either species the causes and selective forces maintaining these different morphotypes remain to be investigated.

Similar to previous work on Malagasy *Crematogaster* (Blaimer 2010), the new species described here have very restricted distributions and suggest adaptation to narrow environmental niches. Madagascar is well known for its highly endemic species assemblages (Goodman and Benstead 2005), but, similar to other biodiversity hotspots in the world, habitat destruction is posing a constant threat especially to these locally endemic taxa. For instance, one of the three localities at which the newly described *Crematogaster mpanjono* occurs is a currently unprotected parcel of littoral forest, Ambohidena on Ile de St. Marie (see Goodman 1993). Littoral forests represent one of the most threatened vegetation types in Madagascar (Consiglio et al. 2006) and forests in Madagascar that are not under protection are inexorably shrinking further (Brooks et al. 2002; Allnutt et al. 2008). Although one new endemic ant species will not be sufficient to motivate protection of forests such as Ambohidena, adding and tallying these numbers across a breadth of taxa, as was achieved recently by Kremen et al. (2008), will aid tremendously in ongoing efforts to define priorities for the expansion of the protected area network in Madagascar. In particular arthropod taxonomists are lagging behind in describing Madagascar’s incredibly diverse fauna. The present study offers a small contribution to fill this gap.

## Supplementary Material

XML Treatment for Crematogaster rasoherinae

XML Treatment for Crematogaster madecassa

XML Treatment for Crematogaster telolafy

XML Treatment for Crematogaster razana

XML Treatment for Crematogaster volamena

XML Treatment for Crematogaster mpanjono
